# Towards Sustainable Utilization of Rejuvenated Bitumen: A Review with Emphasis on Secondary Aging, Aging Resistance, and Multiple Rejuvenation

**DOI:** 10.3390/polym18172103

**Published:** 2026-08-29

**Authors:** Hongbin Zhu, Naisheng Guo, Yuanyuan Li, Shisong Ren, Fu Wang, Jun Zhang, Zhi Zheng, Hang Su, Zenggang Zhao, Ke Zhang

**Affiliations:** 1 College of Transportation Engineering, Dalian Maritime University, Dalian 116026, China; zhuhongbin@dlmu.edu.cn (H.Z.);; 2 School of Civil Engineering and Architecture, Wuhan Institute of Technology, Wuhan 430074, China; 3 Sustainable Pavements and Asphalt Research (SuPAR) Group, Faculty of Applied Engineering, University of Antwerp, 2020 Antwerp, Belgium; shisong.ren@uantwerpen.be; 4 School of Materials and Energy, Central South University of Forestry and Technology, Changsha 410004, China

**Keywords:** bitumen, aging resistance, multiscale evaluation, secondary aging, multiple rejuvenation, rejuvenator

## Abstract

To further advance the recycling of pavement waste, the secondary-aging and multiple-rejuvenation processes of bitumen are first systematically reviewed. Beginning with primary-aging mechanisms, rejuvenator classification and physicochemical properties are summarized to aid elucidation of primary rejuvenation. Secondary-aging evolution of technical and physicochemical properties of rejuvenated bitumen (RB) is critically analyzed. The aging resistance of virgin bitumen and RB is quantitatively compared via extensive literature data. The background and progress of multiple rejuvenation cycles are also outlined. The existing literature shows that identical aging modes can induce distinct component variations, while degradation patterns remain consistent across modes. Among petroleum-based rejuvenators, those with aromatic content exceeding 60% are widely used, and the polar carboxyl groups of tall oil account for their high efficacy. RB aging resistance strongly correlates with both rejuvenator type and dosage. Most RB outperforms virgin bitumen in aging resistance, and post-aging variation amplitudes of softening point and high-temperature failure temperature are approximately 20% for both, while those of penetration and viscosity range from 60% to 120%. Multiple aging–rejuvenation trends resemble those of primary cycles, with progressive macro- and micro-property deterioration as cycles increase. Future work includes developing high-performance bitumen from aged feedstock, establishing molecular screening systems for fatty acid bio-oil rejuvenators, and advancing multiple rejuvenation via molecular dynamics.

## 1. Introduction

Asphalt pavements are widely constructed across the globe due to their favorable inherent properties, especially for high-grade highway and urban road construction [1,2,3]. Nevertheless, bitumen undergoes aging under the combined effects of traffic loading and environmental factors, leading to progressive pavement performance deterioration, reduced durability, and significantly shortened service life [4,5,6,7,8,9].

Asphalt pavements are milled by road maintenance authorities at the end of their service life, whether they fail prematurely or operate normally. To preserve satisfactory pavement performance and operational safety, as much as 70 to 90 million tons of reclaimed asphalt pavement (RAP) is generated annually from road maintenance and rehabilitation projects in China alone [10,11]. Globally, the scale of RAP generation is even more substantial. Europe and the United States collectively produced over 100 million tons of RAP in 2020, with more than 95% reused, while recycling practices have also been widely adopted across Asian countries, including Indonesia, Japan, South Korea, and Malaysia, as well as in Oceania and Africa [12]. Extensive research confirms that RAP incorporation into new asphalt pavements is a highly promising sustainable waste recycling strategy [13,14,15,16,17]. RAP consists of roughly 95% reclaimed aggregate and 5% RAP binder. While bitumen accounts for only 5% of the mixture by mass, it contributes over 60% of total mixture material costs for new pavement construction [18]. It has been widely documented that production costs of asphalt mixtures are reduced by approximately 28% with 50% RAP binder incorporation, and cost savings of 50% to 70% can be achieved at 100% RAP binder content [19]. From an environmental perspective, the demand for new bitumen is effectively reduced through rejuvenation and reuse of RAP binder, and fuel consumption and greenhouse gas emissions are reduced correspondingly [7,9,20]. Quantitatively, life-cycle assessments indicate that RAP incorporation into asphalt layers can reduce the overall environmental impact by 15% to 30%, with greenhouse gas emissions decreasing by up to 17% when virgin aggregate is replaced by 50% [12,21]. Therefore, significant economic, resource, and environmental benefits are delivered by RAP binder recovery and rejuvenation.

Rejuvenated bitumen (RB) produced from RAP binder via rejuvenation technologies has been widely proven feasible for new pavement construction [6,9,13,22]. However, rejuvenated pavements are susceptible to structural damage, low-temperature cracking, and moisture damage, with durability inferior to that of conventional asphalt pavements. Consequently, RB application is strictly limited in high-grade roads, as well as the middle and upper layers of asphalt pavements [23,24]. Accordingly, the long-term performance of RB warrants focused research attention. Notably, RB in service is also exposed to natural environmental factors and traffic loading, which triggers pavement re-aging. This process is defined as secondary aging. It specifically refers to the aging that RB undergoes after it has been restored and returned to service, distinct from the primary aging that occurred during its initial service life [25,26]. Existing studies confirm that secondary aging performance of RB is closely associated with rejuvenator type, dosage, and incorporation method, as well as aged-asphaltene content [13,27,28]. A robust correlation has been reported between rejuvenation performance and subsequent aging behavior of RB [29,30]. Given the current status of secondary aging in rejuvenated pavements, there is an urgent demand for RB re-rejuvenation. Future rejuvenated pavements will inevitably face challenges associated with secondary and subsequent rejuvenation cycles. These cycles are herein termed multiple rejuvenation, which refers to the repeated cyclic process of rejuvenation, in-service re-aging, and subsequent rejuvenation of bitumen over two or more cycles. Encouragingly, a limited number of studies on multiple bitumen rejuvenation have been published to date [31,32,33]. However, due to wide variations in currently adopted rejuvenator types, RB preparation protocols, and secondary aging methods, the performance evolution and underlying mechanisms of RB during secondary aging and multiple rejuvenation have not been systematically summarized or elucidated. Its aging resistance and influencing factors have also not been comprehensively reviewed.

**Figure 1:**
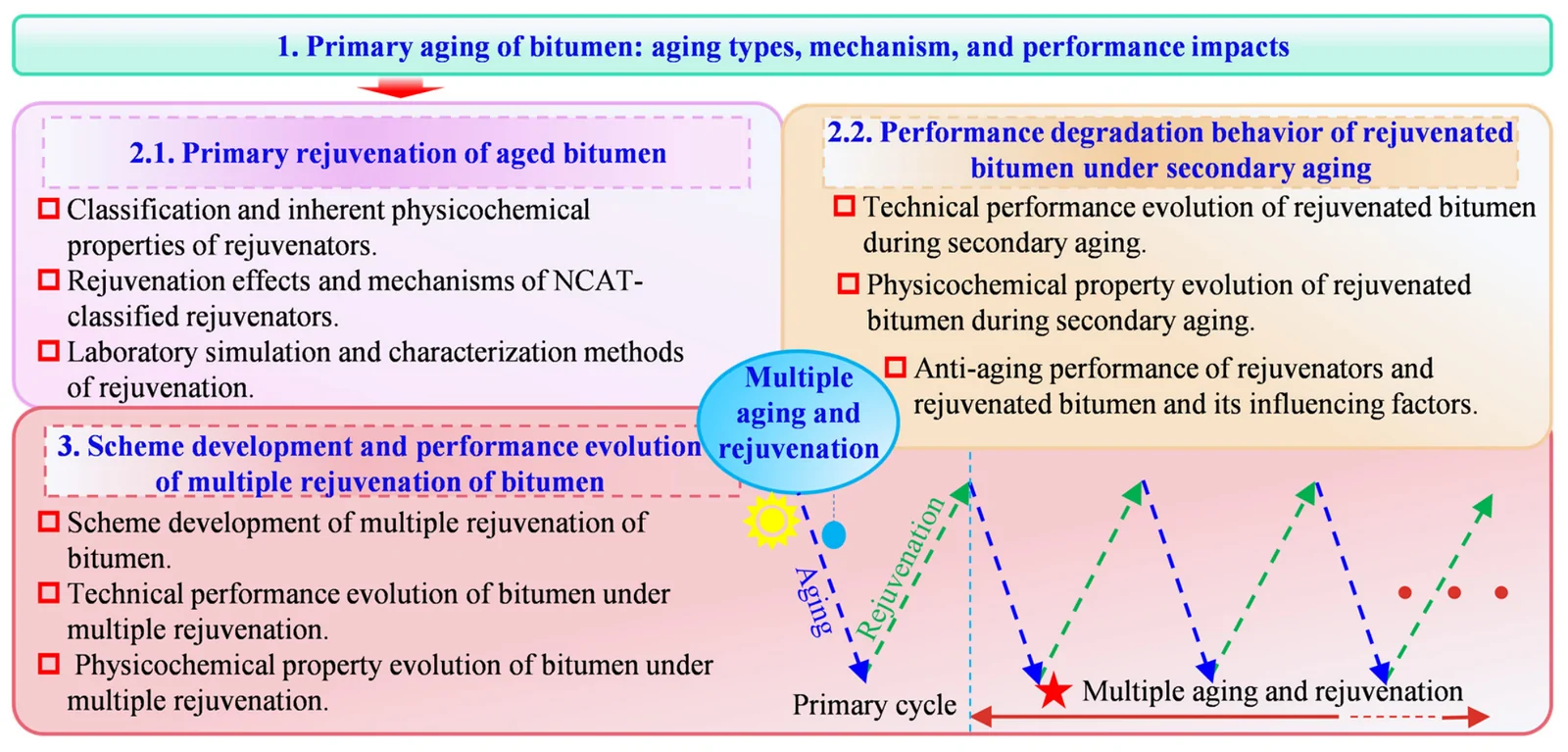
Review map.

The core objective of this review is to analyze limitations of current bitumen secondary aging and multiple-rejuvenation technologies, and outline future research trends and key priorities in this field. Building on global research advances in bitumen aging and rejuvenation cycles, this review first elucidates primary bitumen aging mechanisms and systematically summarizes rejuvenator classification, physicochemical properties, primary-rejuvenation mechanisms, and laboratory simulation and characterization methods for aging and rejuvenation. As illustrated in Figure 1, RB secondary-aging behavior is then comprehensively analyzed across three dimensions covering technical performance, physicochemical properties, and aging resistance. The influencing factors of rejuvenator type and secondary-aging protocols on bitumen macro–micro-properties are clarified, and the underlying regulatory mechanism of RB aging resistance is identified. In response to growing demand for asphalt-pavement recycling, the latest research progress on multiple rejuvenations is further summarized. This review delivers the first systematic synthesis of RB performance evolution and underlying mechanisms across multiple rejuvenations, thereby filling a critical gap in the existing literature. It also provides theoretical support for multi-cycle RAP resource utilization and the green, sustainable development of bitumen-rejuvenation technologies.

## 2. Primary-Aging Mechanism of Bitumen

Three primary-aging mechanisms are recognized for bitumen, including thermo-oxidative aging, ultraviolet aging, and moisture aging [34,35]. Among these, thermo-oxidative aging dominates multi-factor coupled aging processes, owing to its universal mechanism and severe performance-degradation effect [36]. Thermo-oxidative aging is divided into two stages based on occurrence scenario and chronological order. The first stage is short-term aging occurring during pavement construction, and the second stage is long-term aging across the full pavement service life [36,37]. Dynamic superposition of short-term and long-term aging continuously alters bitumen chemical composition, triggering a series of physicochemical reactions, including polymerization, oxidation, and light-component volatilization. These changes ultimately drive bitumen’s molecular structure evolution and component-proportion disequilibrium [38,39,40]. As a typical colloidal material, bitumen is divided into four components: saturates, aromatics, resins, and asphaltenes. Its physicochemical properties are essentially governed by the equilibrium of its four-component system. These four components form a stable colloidal structure via intermolecular interactions that constitutes the microscopic foundation for the macroscopic performance of bitumen materials [41,42,43].

At the microscopic reaction level, the core of bitumen aging is the breakdown of dynamic equilibrium between the destruction and reconstruction of the original colloidal structure [44]. During aging, aromatic C-H bonds in aromatic hydrocarbons are cleaved first, and unsaturated carbon-bond structures form with hydrogen-atom loss [45]. Dehydrogenated aromatic molecules undergo carbon atom sharing via condensation reactions, accordingly generating higher-molecular-weight polycyclic aromatic hydrocarbon structures [46]. This conversion pathway directly drives the migration of aromatics to resins and asphaltenes. This migration disrupts native bitumen colloidal equilibrium and consequently triggers macroscopic performance deterioration [47,48,49]. Meanwhile, oxidation reactions produce oxygen-containing functional groups, including carbonyl and sulfoxide groups, which increase molecular polarity and strengthen intermolecular association. An increase in relative hydrogen atom content of bitumen molecular side chains is observed. Aliphatic side-chain elongation also intensifies internal friction during molecular motion [50]. The synergistic effect of these two mechanisms promotes the formation of higher-molecular-weight aggregates [51,52]. Existing studies also confirm that benzene ring-adjacent groups and unsaturated bond sites show specific oxidation reactivity. Specifically, unsaturated carbon-bond structures formed via dehydrogenative condensation further boost active site reactivity. Chemical-bond recombination at these active sites is widely accepted as the core intrinsic driving force for continuous bitumen microstructure evolution [45,53,54].

**Figure 2:**
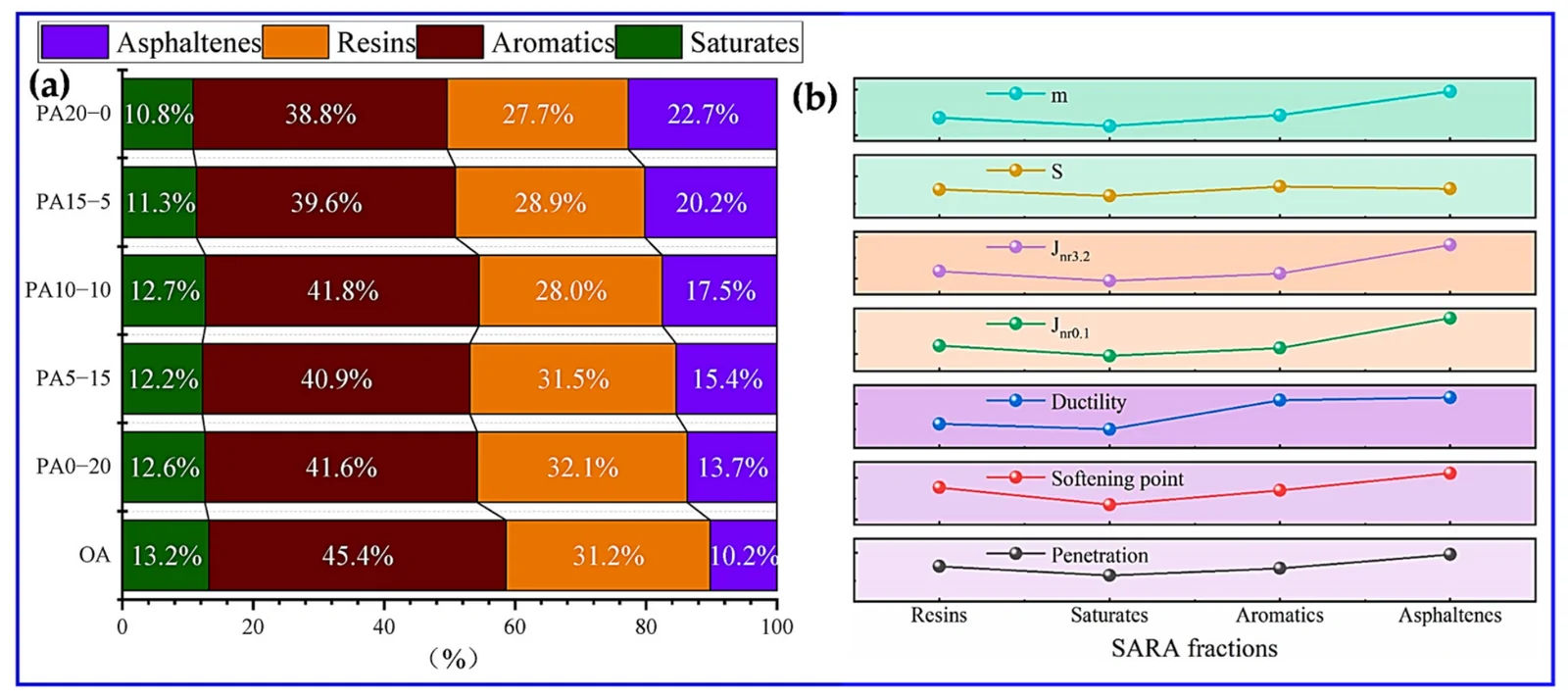
Component results ((**a**) component changes in SMB under water aging and thermal–oxidative aging [55]. (**b**) Influence degree of components on the high- and low-temperature performance of bitumen [56]).

Figure 2 shows the component results. Among them, Figure 2a presents component variations in styrene–butadiene–styrene-modified bitumen (SMB) under water aging and thermal–oxidative aging. Notably, the SMB contains styrene–butadiene–styrene at 5% by mass of the bitumen. The sample designation PA5-15 denotes 5 h of moisture aging followed by 15 h of pressure aging, and so forth. As can be observed, under both moisture aging and thermal–oxidative aging, the asphaltene content of the bitumen increases significantly, whereas the aromatic content decreases significantly. The influence of moisture aging on bitumen composition is markedly stronger than that of thermal–oxidative aging. Figure 2b presents the degree of influence of components on the macroscopic performance of bitumen. It can be observed that the aromatics and asphaltenes significantly affect the macroscopic performance of bitumen, whereas the saturates and resins exert a relatively minor influence on that of bitumen.

Table 1 summarizes the mechanisms of different aging types and their impacts on bitumen properties, corroborating part of the aforementioned conclusions. Light-component volatilization occurs in the early stage of thermo-oxidative aging, followed by a cascade reaction where aromatics convert to resins, and resins further transform into asphaltenes [49,57]. Ultraviolet aging induces molecular chain scission via photochemical reactions, thus accelerating bitumen oxidation and crosslinking [58,59]. Moisture aging includes physical water diffusion and chemical hydrolysis, which promotes coupled thermo-moisture and photo-moisture aging [60,61,62]. A strong correlation exists between macroscopic bitumen property evolution and microstructural changes. This is evidenced by degraded elasticity, permeability, ductility, and rheological behavior, increased softening point and viscosity, and reduced fatigue and cracking resistance of asphalt pavements. While aging moderately improves material high-temperature stability, its adverse impacts on low-temperature cracking resistance and moisture stability are more prominent. These effects directly raise the incidence of early pavement distress, considerably shorten road service life, and reduce riding comfort [63,64,65].

Overall, primary bitumen aging is characterized by component transition from dynamic equilibrium to disequilibrium. The associated physical performance deterioration stems from colloidal structure disruption and polar substance accumulation. Existing bitumen aging studies mainly focus on clarifying single-stage deterioration mechanisms and performance-degradation patterns. The correlation between macro–micro-property variations and aged bitumen (AB) chemical composition changes remains poorly understood. Standardized effective evaluation indicators for bitumen aging are still lacking. Although the conventional short-term/long-term aging classification is well established for virgin bitumen (VB) within a single service life, its application to the multiple aging–rejuvenation framework requires clarification. Specifically, each rejuvenation cycle reintroduces a short-term aging stage during reclaimed mixture construction and a subsequent long-term aging stage throughout the rejuvenated pavement’s service life. Yet the aging object shifts from VB to RB. Primary-aging mechanisms therefore cannot be directly extrapolated to subsequent cycles. In the following sections, secondary aging of RB is defined as the cyclic aging–rejuvenation–re-aging process experienced by RB during service. Beyond performance degradation, core features of this process include accumulation and interaction of VB, AB, and rejuvenators. These interactions make the underlying mechanisms of the full cycle more complex.

## 3. Primary Rejuvenation of AB

### 3.1. Rejuvenator

#### 3.1.1. Classification of Rejuvenators

Currently, bitumen-rejuvenation methods are broadly divided into two categories. The first is achieved by blending with VB or polymer-modified bitumen, and the second relies on rejuvenator modification [72,73,74,75,76]. However, this VB-based rejuvenation strategy requires high VB dosages to blend with small AB proportions, making it difficult to meet current practical AB recycling demands [77,78]. In contrast, rejuvenator-based modification methods offer more prominent advantages, as they restore performance of large AB volumes with only low rejuvenator dosages.

Rejuvenators are defined as substances that restore AB performance to meet standard service requirements, and they typically consist of hydrocarbon mixtures [13,79]. In rejuvenation-mechanism research, rejuvenator classification systems have gradually expanded from molecular structural attributes to functional properties. Multi-dimensional classification frameworks have been developed based on interaction patterns between rejuvenator physicochemical properties and bitumen components. From the molecular configuration perspective, rejuvenator molecular structures are categorized into four classes by Sun et al. [80], including cyclic saturated hydrocarbons, straight-chain saturated hydrocarbons, naphthenic aromatics, and polar aromatics. Based on functional group characteristics, rejuvenators are divided into five classes by Han et al. [81], including fatty acids, amides, phenols, aromatic hydrocarbons, and saturated hydrocarbons. Rejuvenators can also be divided into three functional categories, including softeners for viscosity adjustment, replenishers for light-component supplementation, and emulsifiers for asphaltene depolymerization [82,83].

**Figure 3:**
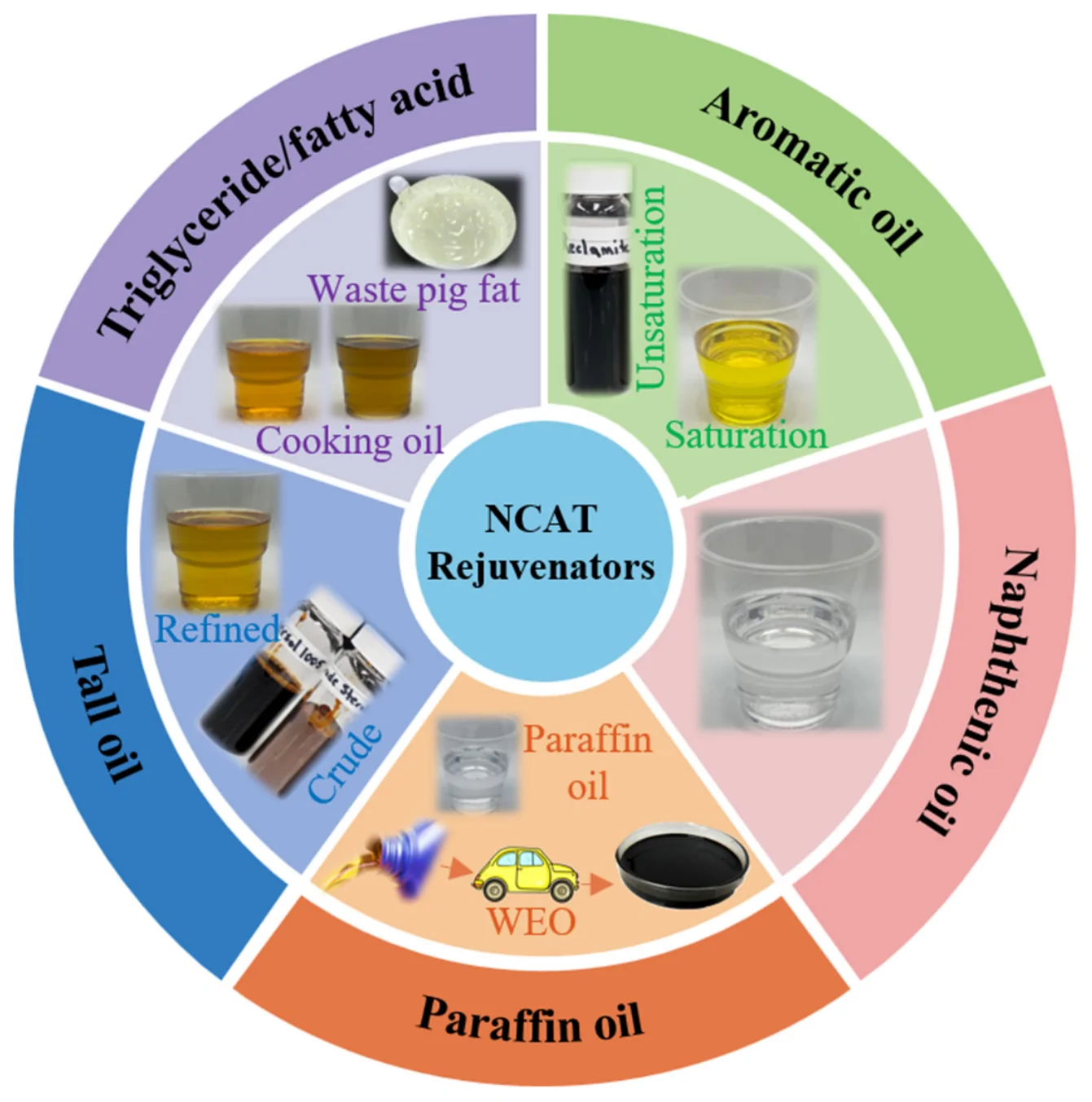
NCAT rejuvenator types [84,85].

The National Center for Asphalt Technology (NCAT) categorizes rejuvenators into five main groups, including aromatic oils, naphthenic oils, triglycerides/fatty acids, paraffinic oils, and tall oils [13,15]. Figure 3 outlines the five NCAT-classified rejuvenator categories. Overall, the NCAT classification system is widely accepted as the most authoritative rejuvenator classification framework. It further differentiates rejuvenators by underlying action mechanisms on the basis of source and sustainability.

**Figure 4:**
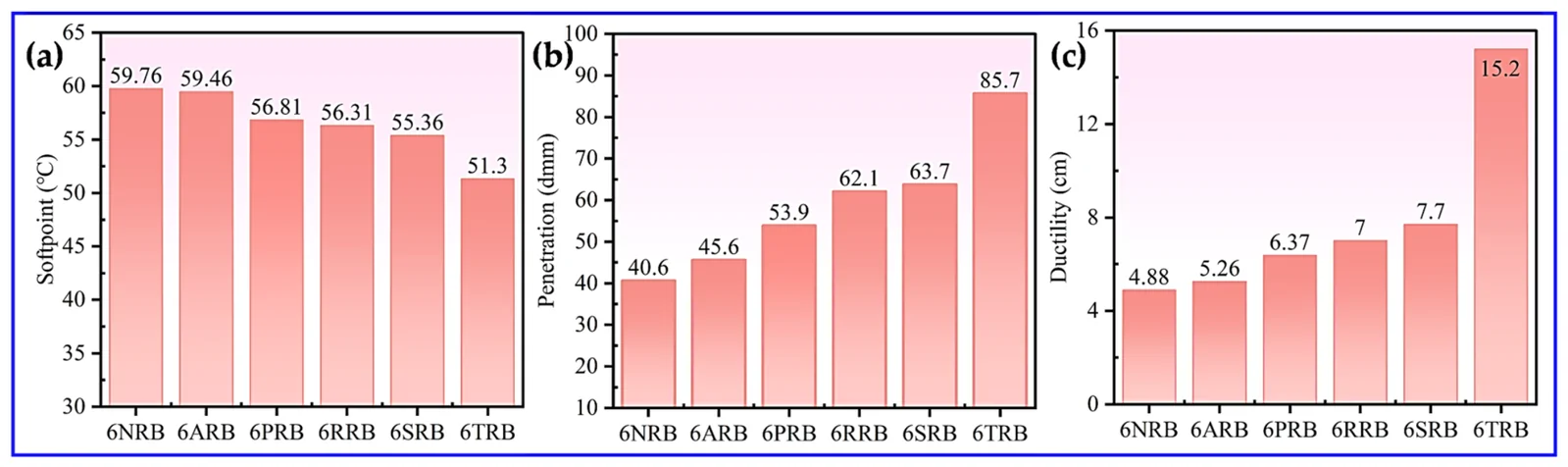
The rejuvenation effect of NCAT rejuvenators on the three major parameters ((**a**) softening point, (**b**) penetration, and (**c**) ductility); these data were obtained from Reference [13].

For rejuvenator compositional requirements, high aromatic content and low saturate content are generally considered critical to improve the gelling capacity of the maltene phase [86]. This is because aromatics effectively dissolve and peptize asphaltenes, whereas saturates are poor solvents for asphaltenes [47]. During rejuvenation, aromatic-rich rejuvenators re-dissolve and re-disperse aggregated asphaltenes in AB via aromatic solvation. This restores the solvation equilibrium of the maltene phase and rebalances AB composition to performance-required proportions, thus recovering technical properties of AB. This criterion is primarily established for highly aromatic rejuvenators and may not apply universally to bio-based and non-aromatic rejuvenators. To further examine the compositional characteristics of various rejuvenators, the four-component ratios of selected rejuvenators are summarized in Table 2. The aromatic content of most rejuvenators exceeds 60%, which is consistent with the conclusion of Dunning et al. [87] that aromatic components in rejuvenators should make up over 60% of total mass. Beyond compositional profiles, the rejuvenation effect of different NCAT-classified rejuvenators also varies considerably, as illustrated in Figure 4. Owing to its abundant polar carboxyl groups, tall oil delivers the highest comprehensive efficiency among all rejuvenators, followed successively by soybean oil, rapeseed oil, paraffinic oil, aromatic oil, and naphthenic oil. In light of the four-component data presented in Table 2, the efficiency differences among these rejuvenator categories can be partly attributed to their varying aromatic and saturate contents. Rejuvenators with higher aromatic fractions and lower saturate fractions generally possess stronger asphaltene dissolution capacity. This capacity, combined with active polar functional groups, explains the superior performance of tall oil-based and triglyceride/fatty acid-based rejuvenators relative to that of paraffinic and naphthenic oils. Although aromatic oils have high aromatic content, their composition closely resembles that of the native aromatics in AB. The absence of additional polar functional groups results in only moderate rejuvenation efficacy. With respect to asphaltene content, asphaltenes are rarely detected in rejuvenators. This feature promotes AB dilution by rejuvenators, leading to a rapid drop in AB asphaltene content. Building on these compositional principles, conventional rejuvenating materials widely used include aromatic oils [88,89,90], waste cooking oil (WCO) [16,17,68,91], waste engine oil (WEO) [27,47,92], and commercial rejuvenators [31,75,93,94].

#### 3.1.2. Physicochemical Properties of Rejuvenators

(1) Aromatic oils

Aromatic oils are a category of rejuvenators consisting mainly of aromatic hydrocarbons, with an average chemical formula of C_30_H_40_ reported in the literature [82,105,106]. Reported properties of aromatic oils include a molecular weight of approximately 409.99 g/mol, a density of around 1.05 g/cm^3^ [88,107], and a solubility parameter around 19 J/cm^−3/2^ [108,109]. These density and solubility parameters are indicative of favorable compatibility between aromatic oils and AB (density ranges from approximately 1.05 to 1.08 g/cm^3^ [16,17,110,111,112,113]). From an environmental perspective, aromatic oils are classified into conventional high-aromatic oils and saturated aromatic hydrocarbon-based types. Conventional high-aromatic oils contain carcinogenic components, including polycyclic aromatic hydrocarbons with potential health risks. Relatively high dosages are therefore needed to reach target performance [82,114,115]. Notably, aromatic oils are frequently adopted as a benchmark to evaluate the rejuvenation efficacy of other rejuvenators [82,114].

(2) Naphthenic oils

Naphthenic oils are organic solvents derived from naphthenic base crude. They feature high density, high viscosity, non-toxicity, and low cost, with an average chemical formula of C_26_H_48_ reported in existing studies [96,105]. Documented properties of naphthenic oils include a molecular weight of 357.06 g/mol and a solubility parameter of 17.562 J/cm^−3/2^ [109]. No heteroatomic functional group is found in the molecular structure of naphthenic oils. Due to their polycycloalkane structure, the intermolecular potential energy of naphthenic oils is lower than that of aromatic oils and bio-oils, but higher than that of engine oils [105].

(3) Triglycerides/fatty acids

A broad range of materials fall under triglyceride/fatty acid-based rejuvenators, including bio-oils (average molecular formula of C_19_H_36_O_2_ [105]), vegetable oils, WCO, soybean oil, animal fat oils, oils derived from pig manure, and epoxidized bio-oils [82,116,117]. Fatty acids are recognized as the primary components of these rejuvenators, with molecular structures composed of alkane chains and polar carboxyl end groups. Fatty acids are divided into saturated and unsaturated categories [118]. Unsaturated fatty acids exhibit higher reactivity and molecular polarizability due to the presence of C=C bonds [106,119]. In contrast, saturated fatty acid content must be strictly regulated due to paraffin-like crystallization properties to avoid degradation of RB low-temperature performance [120]. During fatty acid oxidation, oxidation reactions are readily induced by the C=C bonds in unsaturated fatty acids. Carbonyl groups, aldehydes, and high-molecular-weight compounds are generated as by-products [106,121,122,123].

(4) Paraffinic oils

Paraffinic oils are petroleum by-products from lubricant refining, consisting mainly of saturated hydrocarbons with structures similar to saturated carbon chains in bitumen [124,125,126]. WEO, waste engine-oil bottoms (WEOBs), and waste hydraulic oil are all recognized as common paraffinic oil-based rejuvenators [82,98,106]. One study reports a molecular weight of 316.48 g/mol and a solubility parameter of 16.890 J/cm^−3/2^ for engine oil [109]. Previous studies have documented poor compatibility of paraffinic oils with low-polarity naphthenes and polar bitumen components. This incompatibility frequently causes colloidal stability problems [124,125]. At high crystallization temperatures in particular, paraffinic oils readily induce asphaltene precipitation and colloidal instability, an effect more prominent in deeply AB [126,127]. This constitutes one of the factors limiting its potential as a high-performance rejuvenator.

(5) Tall oils

Tall oil is a by-product of pine pulping in the paper industry, extracted from residual black liquor after pulping. It has a complex heterogeneous chemical composition, consisting mainly of fatty acids; rosin acids; and unsaponifiable matters, such as sterols, waxes, and hydrocarbons [13]. The fatty acid component is dominated by approximately 46% oleic acid, 48% linoleic acid, and 6% palmitic acid. Exact fatty acid composition depends on multiple factors, including wood species, pulping methods, storage conditions, and regional variability [128,129,130,131]. Asphalt mixtures prepared with high-resin crude tall oil show lower rutting resistance than those with distilled tall oil. Distilled tall oil performs better due to its lower resin content. This effect is attributed to resin conversion to asphaltenes during long-term aging, which reduces fatigue resistance [106,132,133].

### 3.2. Rejuvenation Effects and Mechanisms of Rejuvenators

#### 3.2.1. Rejuvenation Effects of Rejuvenators Based on NCAT Classification

(1) Aromatic oils

Aromatic oils are classified as replenishment-type bitumen rejuvenators. Their core rejuvenation mechanism involves compensating for saturated and aromatic components lost in AB. Partial depolymerization of asphaltene aggregates is achieved through weak intermolecular polar interactions [82,114,126,134]. Owing to chemical structures similar to bitumen saturates and aromatics, aromatic oils show good miscibility with AB and effectively replenish light components lost during aging [53]. As typical petroleum-based rejuvenators, aromatic oils have been proven to effectively enhance AB fatigue and low-temperature properties. They also impart favorable aging resistance to the resulting RB [63,135]. Meanwhile, their molecular structures contain no hydrophilic group, endowing the prepared RB with excellent moisture-damage resistance [136]. However, the relatively low polarity of aromatic moieties in aromatic oils results in limited interaction strength with asphaltene fused-ring structures [42,126,134]. Molecular dynamics studies have also confirmed that fused aromatic rings in aromatic oils readily form strong intermolecular forces via π-π stacking. The highly compact and stable asphaltene aggregate structure derived from such strong interactions impedes the penetration of rejuvenator molecules into the internal stacked structure. This accounts for their limited depolymerization efficiency [54,80,105,137,138]. Additionally, effective rejuvenation with aromatic oils requires high dosages. Furthermore, partial aromatic hydrocarbons readily oxidize into resins during mixing, reducing the actual content of effective rejuvenating components [114,115]. To mitigate these limitations, AB rejuvenation efficiency of aromatic oils can be further improved by blending with plasticizers [139,140].

(2) Naphthenic oils

Naphthenic oils can effectively restore AB chemical composition, reducing colloidal index and recovering physicochemical properties to levels comparable to base bitumen. Long-term rutting and fatigue performance stability of RB can be maintained, delivering generally good long-term service stability [141]. However, colloidal index rebound frequently occurs in naphthenic-oil RB during long-term aging. This is mainly attributed to asphaltene precipitation caused by excessive saturated hydrocarbon content in the system [142,143]. Currently, no academic consensus has been reached on the compatibility of naphthenic oils with bitumen systems. Some studies report that high saturate content in naphthenic oils causes poor bitumen compatibility [63,135]. Others show that naphthenic oils can improve compatibility between phase change materials and bitumen [144]. In terms of working efficiency, the rejuvenation and diffusion efficiencies of naphthenic oils are moderate. These efficiencies have been proven to be lower than those of bio-oils and engine oils but higher than those of aromatic oils. Their effective action surface area exceeds that of aromatic oils and bio-oils [105,145].

(3) Triglycerides/fatty acids

The core rejuvenation effect of triglyceride/fatty acid-based rejuvenators stems from asphaltene aggregate dispersion and depolymerization. Their performance is closely tied to their molecular structural features [13,15]. Unsaturated fatty acids disperse asphaltene aggregates via C=C bond polarization. Free fatty acid (FFA) carboxyl groups further strengthen this effect, enabling rapid AB depolymerization [13]. Saturated fatty acid content must be kept low to avoid crystallization and precipitation that degrade the rutting resistance and low-temperature performance of RB [120]. Notably, while high unsaturated fatty acid content boosts rejuvenation efficiency, its oxidation-prone property reduces fatigue resistance of RB. The saturated fatty acid content must therefore be properly balanced in practice [106].

In terms of intrinsic molecular interaction mechanisms, the molecular potential energy of these bio-oils is higher than that of engine and naphthenic oils. This is mainly due to the hydrogen-bonding capacity of their polar ester groups. At extremely low temperatures, bio-oil molecules readily aggregate due to polar ester group interactions. Meanwhile, bio-oils exhibit the highest molecular mobility. Aromatic oils and engine oils have more stable molecular configurations due to greater structural stability [105]. While both bio-oils and aromatic oils undergo molecular aggregation, their underlying driving mechanisms differ fundamentally. Bio-oil aggregation is dominated by hydrogen bonding between polar ester groups. That of aromatic oils arises from aromatic ring π-π stacking [105,146,147].

(4) Paraffinic oils

Paraffinic oils are classified as softening-type bitumen rejuvenators. These materials only serve to soften bitumen and cannot effectively adjust asphaltene aggregate size or morphology [148]. Compared with other rejuvenator categories, paraffinic oils significantly improve AB rheological properties. They also reduce system carbonyl content via a dilution effect immediately after incorporation [149]. However, constrained by high saturated hydrocarbon content and lack of asphaltene depolymerization capacity, RB shows relatively poor colloidal stability after long-term aging. Both aging resistance and durability show marked degradation [142,143].

(5) Tall oils

Tall oils are functionally classified as emulsifier-type bitumen rejuvenators. They feature high light-component content and low viscosity. Their molecules contain strong polar groups, including carboxyl and hydroxyl groups, replicating the rejuvenation mechanisms of bio-oils and modified vegetable oils. Additionally, tall oils exhibit good compatibility with AB due to their small molecular size [82,128,136]. Their rejuvenation effect is achieved via multiple pathways. The carboxyl groups of FFA rapidly depolymerize aged asphaltene aggregates via polar interactions, reducing system viscosity and restoring rheological properties [53]. Meanwhile, polymer–bitumen dispersion is improved via a dissolution effect. These rejuvenators also provide antioxidant functionality, boosting low-temperature cracking resistance of RB [130,131,150]. The depolymerization effect is strengthened by reduced aggregate size and fewer molecules involved in aggregation, with action intensity increasing with dosage. Quantitative studies show that the degree of aged asphaltene aggregation can be reduced by 56.3%, and average molecular stacking distance increased by 35.9% [13,108]. Overall, tall oils effectively restore AB microstructure and mechanical properties at appropriate dosages, showing strong application potential for bitumen rejuvenation [13]. However, tall oils increase RB sensitivity to physical hardening after oxidation. The high hydrophilicity of their molecules markedly raises mixture moisture sensitivity, leading to reduced strength and durability [136,151].

Existing studies have verified favorable comprehensive rejuvenation performance for triglyceride/fatty acid and tall oil-based bio-rejuvenators. Both types achieve efficient asphaltene aggregate depolymerization via fatty acid-induced depolymerization and softening effects. With advances in multi-scale research methods, molecular dynamics simulation is widely recognized as a key tool for uncovering rejuvenators’ microscopic mechanisms [15]. Three main molecular models are used in current bio-oil rejuvenator simulation studies, including methyl esters, FFA, and triglycerides. Molecular models adopted for bio-oils in representative studies are summarized in Table 3. Notably, as a widely studied bitumen rejuvenation material, WCO shows high diversity in molecular model selection, with all three fatty acid forms in common use [16,17,152,153,154,155]. However, a widespread mismatch between model configurations and actual material properties is found in the existing research. For instance, triglycerides are the overwhelmingly dominant component of commercial edible oil, the precursor of WCO, as they ensure storage stability and safety during production. After cooking, only a small share of triglycerides hydrolyzes into FFA, and triglycerides remain the main component of the total system [15]. Triglyceride models should therefore be used for related studies. A study by Ruan et al. [155] was conducted using low-acid-value waste vegetable oil. The low acid value confirms very low FFA content in the system, which is inherently inconsistent with FFA-dominated model assumptions.

This discrepancy demonstrates that bio-oil fatty acid components are simplified to FFA in most studies. The actual presence of ester configurations and their functional differences are largely overlooked, frequently causing systematic biases in rejuvenation-mechanism interpretation. During molecular dynamics simulation and performance prediction, direct application of FFA model parameters to triglyceride-dominated components will overestimate overall rejuvenator system polarity and hydrogen-bond interaction strength. Such overestimation of intermolecular interactions directly distorts the modeled binding affinity and diffusion behavior between rejuvenators and asphaltenes. This thereby undermines the reliability of molecular models for guiding rejuvenator dosage optimization and macroscopic performance prediction. This not only creates ambiguity in molecular-scale rejuvenation-mechanism understanding, but also substantially reduces performance-prediction accuracy.

#### 3.2.2. Rejuvenation Mechanisms of Rejuvenators

As outlined in the rejuvenator classification in Section 3.1, a wide range of rejuvenator types are currently available, with different rejuvenators employing distinct mechanisms to restore AB properties. Existing studies confirm that the rejuvenation process involves mechanisms including physical dissolution, chemical depolymerization, and molecular lubrication. These mechanisms restore bitumen properties across multi-scale levels from molecular to macroscopic. Rejuvenation mechanisms of different rejuvenators and their effects on bitumen’s technical properties are summarized in Table 4. Most rejuvenators achieve AB rejuvenation via asphaltene penetration, softening, and dilution, combined with other auxiliary mechanisms.

Primary bitumen rejuvenation mechanisms have been preliminarily clarified in existing research. As RB multi-cycle recycling is considered in practical engineering, the performance and underlying mechanisms of bitumen during multiple rejuvenations can also be investigated based on primary-rejuvenation theories. However, bitumen under multiple rejuvenations experiences repeated aging and rejuvenation cycles. Rejuvenators and bitumen components of varying aging degrees accumulate in the RB system, making interfaces between different components more complex. Consequently, multiple-rejuvenation mechanisms differ from those of primary rejuvenation and exhibit greater complexity. Multiple-rejuvenation mechanisms remain to be systematically explored.

### 3.3. Laboratory Aging and Rejuvenation Simulation and Characterization Methods for Rejuvenation

#### 3.3.1. Laboratory Preparation Methods

In laboratory conditions, short- and long-term AB are typically simulated via the rolling thin-film oven test (RTFOT) or thin-film oven test (TFOT) coupled with the pressure-aging vessel (PAV) test, yielding laboratory-prepared AB [7,8,13]. AB and RB preparation methods used in multiple-rejuvenation studies by researchers are summarized in Table 5. As shown in Table 5, significant variations exist in shear rate, shear temperature, and shear time selection across different studies. However, test conditions for AB rejuvenation and each RB aging cycle are relatively consistent, typically including RTFOT, TFOT, PAV, and oven-aging protocols. A few exceptional cases are also noted in the literature review. For instance, Ongel et al. [172] used a rotary evaporator for uniform mixing of bitumen and low-viscosity rejuvenators, operating at 65 ± 5 rpm for 5 min at 145 °C and ambient pressure. Mohammadafzali et al. [173] adopted an accelerated pavement weathering system to simulate RB aging over 7 to 10 years. Secondary aging is performed by Li et al. [174] via a modified extended TFOT procedure. When conducting multiple rejuvenation cycles, researchers account for varying aging effects of each cycle on bitumen. Rejuvenator dosage must therefore be adjusted for each rejuvenation step. Three rejuvenation cycles are performed by Chen et al. [32], with rejuvenator dosages of 6%, 10%, and 14% for the first, second, and third cycles, respectively. Blomberg et al. [175] calculated the required rejuvenator dosage for each of four cycles based on the equal penetration criterion. The effect of mixing methods on rejuvenation efficacy has also been explored by researchers, who conclude that mixing methods have limited influence on rheological properties of RB [176].

Overall, no standardized protocol exists for laboratory simulation of multiple aging. RB, like VB, undergoes short-term aging during construction and long-term aging throughout pavement service life. As summarized in Table 5, many studies employ RTFOT/TFOT for short-term aging, followed by a 20 h PAV test for long-term aging. Building on this consensus, each aging cycle in multi-cycle studies should uniformly adopt the RTFOT/TFOT plus 20 h PAV protocol. This aligns with current Superpave specifications. Nevertheless, laboratory accelerated aging compresses years of field aging into tens of hours via elevated temperature and pressure. While it reproduces the overall aging trend at the macroscopic level, it cannot fully capture the complex time-dependent mechanisms driven by coupled diurnal temperature cycles, ultraviolet radiation, moisture damage, and traffic loading under field conditions.

#### 3.3.2. Characterization Methods for Rejuvenation Effect

**Figure 5:**
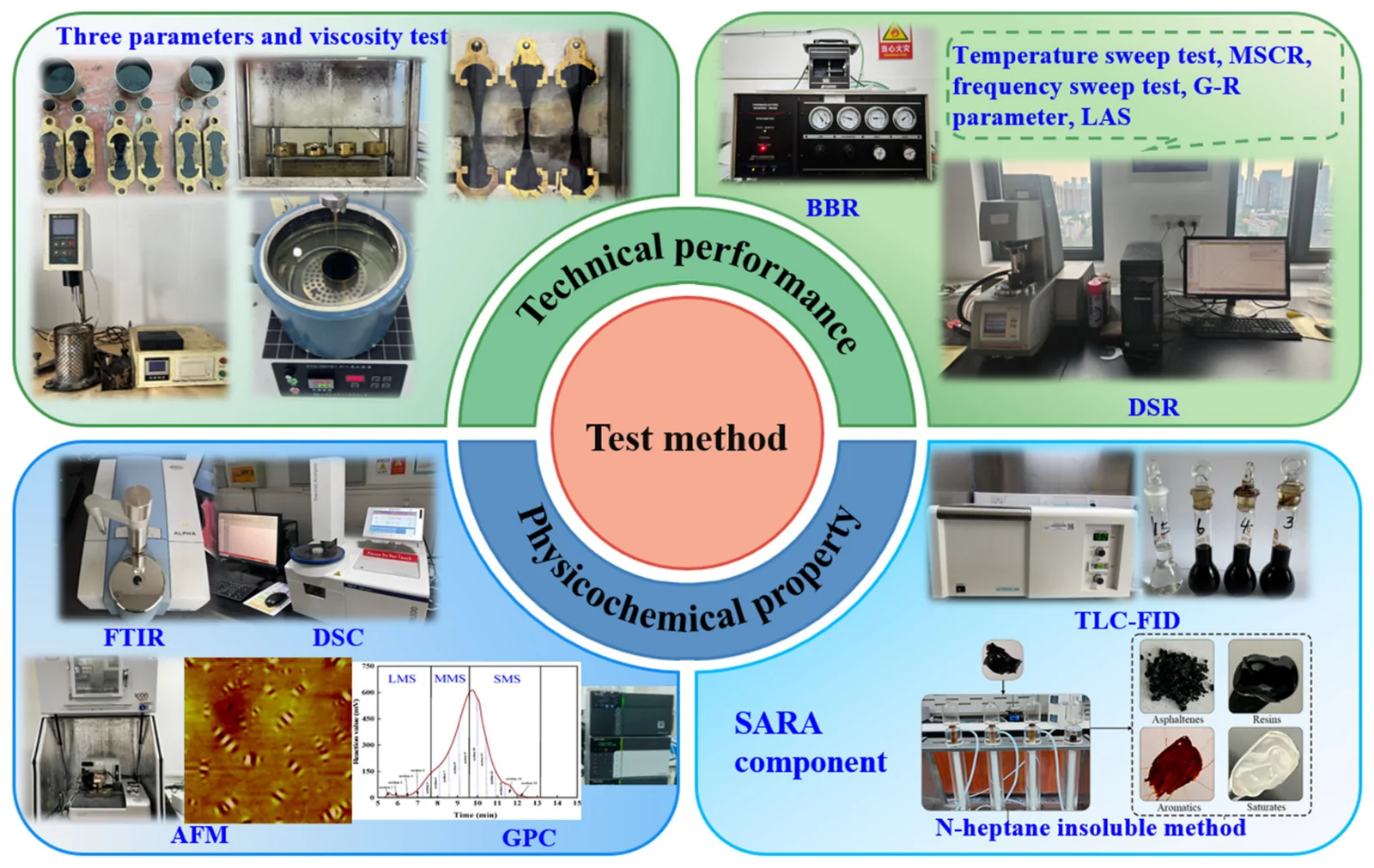
Summary of main test methods for bitumen aging and rejuvenation cycle studies [32,192,193].

Figure 5 outlines the core test methods used in bitumen aging and rejuvenation cycle studies. In bitumen aging and rejuvenation cycle studies, bitumen physical-property evolution before and after each cycle is generally evaluated via viscosity tests, three basic parameter tests, dynamic shear rheometer (DSR), and bending beam rheometer (BBR) tests [13,29,68,188]. Bitumen’s cracking tendency can be assessed using Glover–Rowe (G-R) parameter black space diagrams [30]. Additionally, bitumen’s rutting and fatigue resistance can be further evaluated via the multiple stress creep recovery (MSCR) and linear amplitude sweep (LAS) tests, respectively [74,189,190]. A suite of macro and micro tests is also used to analyze variations in bitumen chemical composition, structure, micromorphology, glass transition temperature (T_g_), and molecular weight distribution before and after each cycle. These tests also help clarify underlying rejuvenation mechanisms. These include thin-layer chromatography with flame ionization detection (TLC-FID), the n-heptane insolubility method, Fourier-transform infrared spectroscopy (FTIR), atomic force microscopy (AFM), differential scanning calorimetry (DSC), and gel permeation chromatography (GPC) [91,98,99]. Common rejuvenation efficiency indicators in existing studies include complex modulus, high-temperature master curve area, crossover modulus, crossover frequency, carbonyl index (CI), sulfoxide index (SI), and aromaticity index [27,68,145]. Crossover modulus and crossover frequency are widely recognized as the most critical indicators for evaluating AB rejuvenation efficiency in related studies [18,27,145,191]. As primary-rejuvenation performance has been comprehensively reviewed in the existing literature, primary-rejuvenation performance and mechanisms are not further discussed in this review.

## 4. Performance-Degradation Behavior of RB During Secondary Aging

### 4.1. Evolution of Technical Properties of RB During Secondary Aging

**Figure 6:**
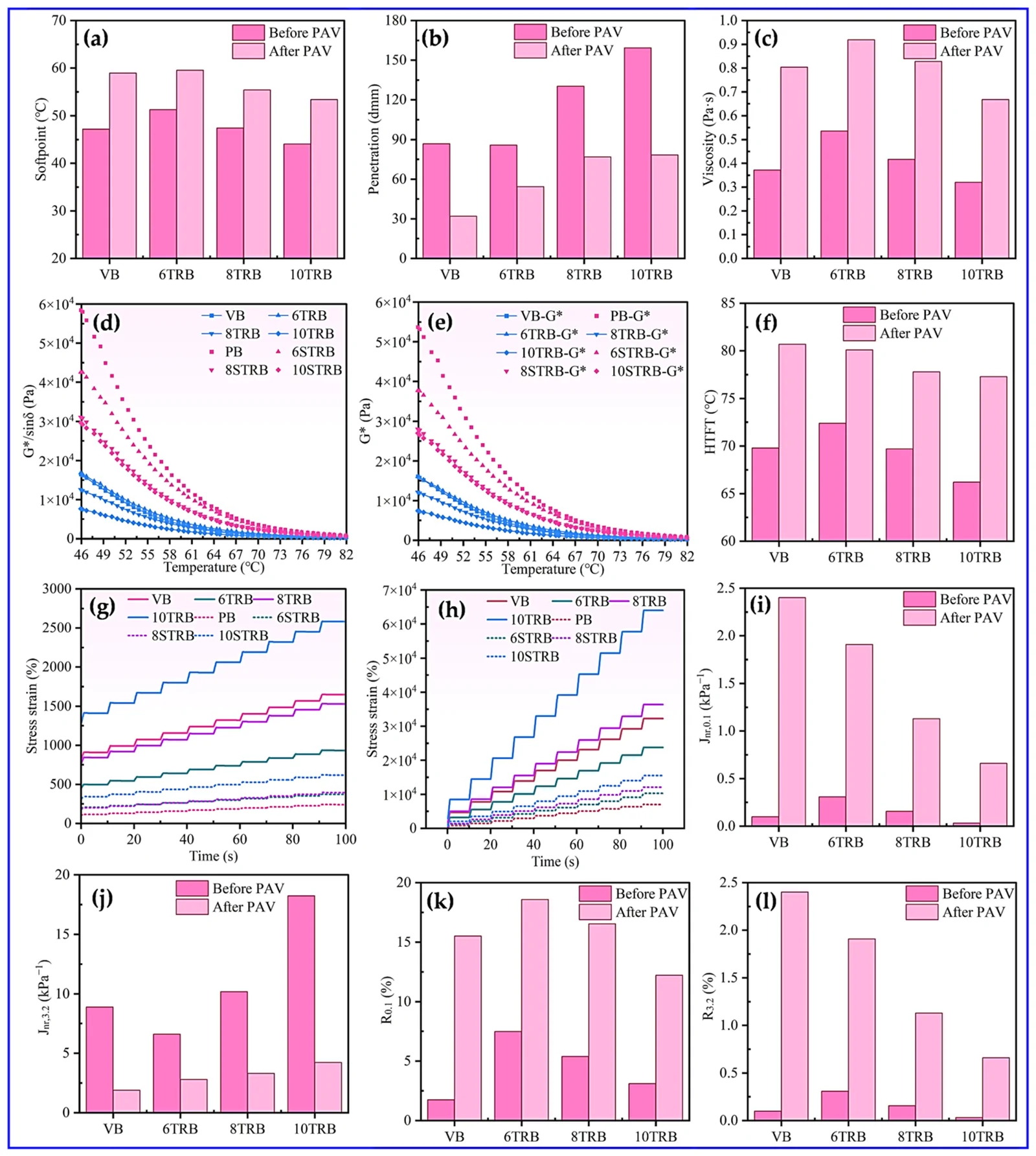
Evolution results of technical properties of RB during secondary aging ((**a**–**c**) basic physical properties, (**d**–**f**) high-temperature temperature-sweep results, and (**g**–**l**) MSCR results); these data were obtained from Reference [13].

Most studies indicate that the evolution pattern of basic physical properties of RB after secondary aging reverses that of the primary-rejuvenation process and parallels the general trend of primary aging, as further discussed in Section 4.2. Specific manifestations include increased viscosity, softening point, complex modulus (G*), rutting factor (G*/sinδ), fatigue factor (G*·sinδ), and damage factor (*D_f_*), as well as synchronous decreases in penetration, ductility, phase angle (δ), non-recoverable creep compliance (*J_nr_*), creep recovery rate (R), and number of cycles to failure life (*N_f_*) [13,25,98,167]. Figure 6 presents the results of our previous study on the technical property evolution of RB during secondary aging. The increases in softening point, viscosity, and high-temperature failure temperature (HTFT), together with the decreases in penetration and *J_nr_*, follow the same pattern documented in existing studies. Additionally, long-term aging shows obvious heterogeneous effects across different indicators, with ductility attenuation significantly greater than that of penetration and softening point [98]. After RB aging, high/low-temperature performance, fatigue performance, and rheological property evolution trends largely reverse those of AB rejuvenation and mirror primary aging [29,92,104,115,176,178,188,191]. Benefiting from the repairing effect of rejuvenators on the rheological properties and cracking resistance of binders, the high-temperature rheological performance of secondary-aged RB is always superior to that of directly secondary-aged raw AB [92,191]. Studies conducted by Asadi et al. [194], Masad et al. [180], and Yan et al. [99] all confirm that rejuvenators effectively enhance RB aging resistance and fatigue performance, slowing performance degradation during aging.

For low-temperature performance, RB exhibits increased stiffness (S) and reduced m-value after aging. Stress relaxation capacity and low-temperature cracking resistance decrease accordingly. Rejuvenator addition can improve secondary-AB low-temperature performance [174,180,181,188]. Luo et al. [98] report that low-temperature performance degradation of WCO- and WEO-rejuvenated RB during secondary aging mainly occurs in the secondary short-term aging phase. Long-term aging of varying durations has relatively limited weakening effects on low-temperature performance. Contradictory findings are reported by other researchers, who argue that rejuvenator addition has no significant effect on the attenuation amplitude of low-temperature performance of RB after secondary aging [124]. No consensus has been reached in existing research on the relative magnitude of short- and long-term aging effects on low-temperature performance.

### 4.2. Evolution of Physicochemical Properties of RB During Secondary Aging

#### 4.2.1. Physical Properties

In AB primary-rejuvenation micromorphology studies, changes in bee-like structure size after bitumen rejuvenation are widely observed via AFM [2,87,171]. After AB rejuvenation, bee-like structure size in RB decreases steadily. A positive correlation exists between bee-like structure size reduction and rejuvenation degree [6,195]. Progress has been made in recent studies on RB secondary-aging micromorphology. For instance, Abdelaziz et al. [115] investigated RB micromorphology under different aging conditions using AFM. Results show that RB contains a matrix phase and an associated phase. The associated phase area or content increases after RB aging, alongside rougher surface texture. An increase in Derjaguin–Muller–Toporov (DMT) modulus is also reported, confirming the correlation between the associated phase and bitumen stiffness. Masad et al. [180] report that further laboratory aging has no significant effect on RAP binder microstructure in RB. This finding aligns with aforementioned chemical structure conclusions. It further validates the conclusion from conventional physical and rheological studies.

**Figure 7:**
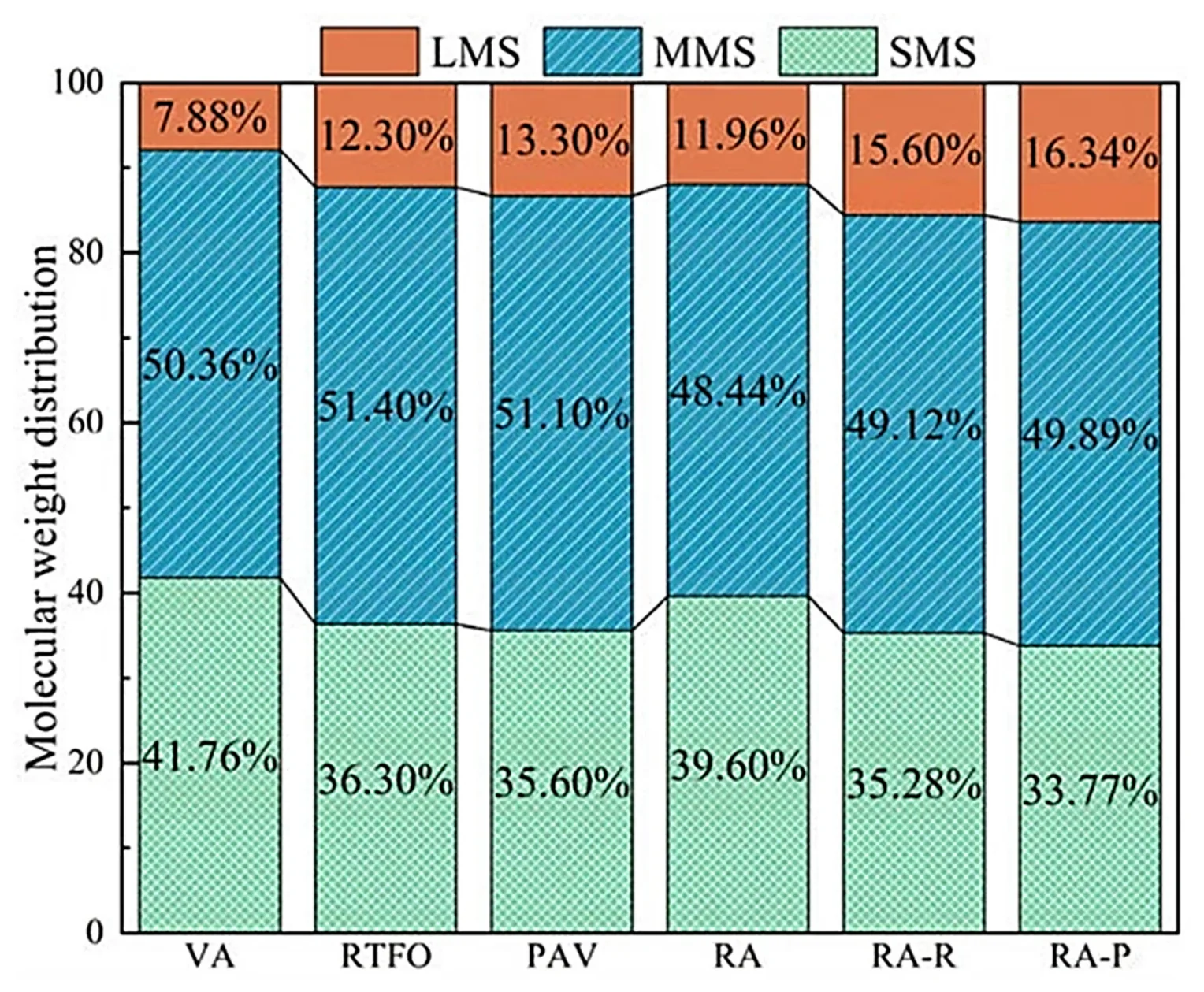
Evolution of relative molecular weight of RB during secondary aging [197].

In primary-aging and -rejuvenation molecular-weight-distribution studies, oxidation converts bitumen molecules to more polar forms, triggering nano-aggregation of oxidized asphaltene molecules. During rejuvenation, rejuvenators depolymerize oxidized asphaltene molecules and restore AB molecular size distribution. Consequently, large molecule content decreases [69,126,168,196]. Bitumen performance is widely recognized to be closely correlated with large molecular species (LMS) [69,99,196]. Similarly, the molecular weight distribution of RB during aging has also been investigated. It is found that the LMS weight-average molecular weight (M_W_) of RB continues to rise after secondary aging, and exceeds that after primary aging [99,176,178,197]. Figure 7 presents the relative molecular weight evolution of RB during secondary aging. This confirms that the proportion of LMS in RB continues to increase after secondary aging. The variation pattern of T_g_ of RB before and after rejuvenation has also been discussed by scholars. It is revealed by T_g_-related studies that aging induces the transition of bitumen from a tough state to a brittle state. An increase in T_g_ value is observed accordingly, which renders bitumen susceptible to cracking. After rejuvenation with the addition of rejuvenators, the T_g_ of AB decreases. The adverse effects of aging on brittleness and low-temperature performance are attenuated [93,177]. After secondary aging of RB, T_g_ continues to rise and exceeds that after primary aging [99,181].

#### 4.2.2. Chemical Properties

**Figure 8:**
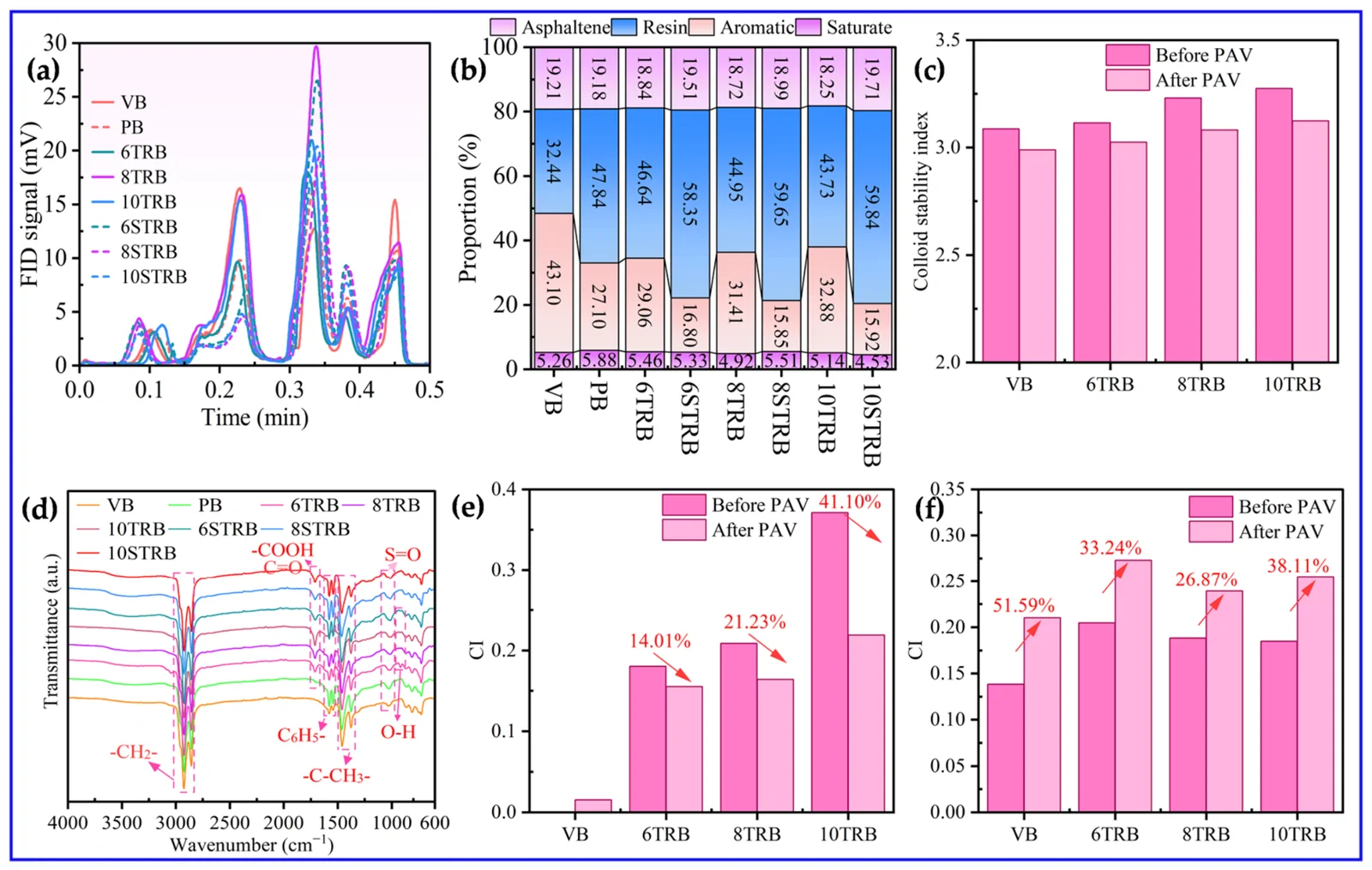
Evolution of chemical properties of RB during secondary aging ((**a**–**c**) chemical components and (**d**–**f**) chemical structures); these data were obtained from Reference [13].

Figure 8 presents the evolution of chemical properties of RB during secondary aging. For chemical composition evolution, rejuvenators supplement light components to AB during rejuvenation. This reduces asphaltene content and restores AB colloidal structure to achieve bitumen performance recovery [6,9,47,68]. The colloidal stability of bitumen is typically characterized by the colloidal stability index, as illustrated in Figure 8c. It plays a critical role in determining its long-term performance and aging resistance. Within a certain range, a higher index indicates a more stable bitumen colloidal system. This generally corresponds to better low-temperature performance and fatigue resistance than those of bitumen with lower colloidal stability. Nevertheless, excessively high values may impair high-temperature performance due to insufficient asphaltene content [6,68]. After secondary aging, relevant studies report increased asphaltene content, reduced light components, and progressive performance degradation [13,98,198]. The changes in bitumen components following secondary aging can be directly observed from Figure 8a–c. Luo et al. [98] found that the colloidal structure of RB is relatively stable, and secondary-aged RB exhibits favorable performance. However, secondary-aging performance does not improve indefinitely with higher colloidal stability. It is also affected by residual components.

In RB secondary-aging chemical-structure studies, it is widely recognized that both CI and SI increase after RB secondary aging. This trend matches primary-aging patterns, confirming that secondary aging is also a chemical process [13,68,199]. Meanwhile, the variation amplitude of CI and SI of AB during secondary aging is generally smaller than that of VB and primary RB. This is attributed to AB having already undergone one rapid aging cycle, resulting in lower aging sensitivity [27,178]. This finding also corroborates the conclusion from conventional physical and rheological studies that AB performance variation amplitude during secondary aging is generally smaller than that of VB and primary RB. The growth rate of CI and SI during the aging process of RB can be effectively slowed down by specific rejuvenators due to their unique chemical composition and structure. These rejuvenators thus present more favorable aging resistance [89,179]. On the contrary, light components in some rejuvenators are susceptible to aging, which accelerates the oxidation process and further increases the aging rate of RB. This also indirectly confirms that secondary aging is a chemical process [27,92,200]. Inherent properties of certain rejuvenators can also accelerate this process. For instance, Zhu et al. [13] reported that for RB made with FFA-based rejuvenators, CI decreases while SI keeps increasing after secondary aging, as shown in Figure 8e,f.

Existing studies consistently demonstrate that the variation trends of technical properties, micromorphology, chemical structure, and relative molecular weight of RB following secondary aging largely match those of VB during primary aging. The abundant aged asphaltenes in AB reduce its aging sensitivity. This thereby yields a performance variation amplitude after secondary aging that is comparatively lower than that of VB. This pattern holds across multiple rejuvenator types and aging protocols, as jointly corroborated by studies spanning multiple dimensions, including rheological properties, micromorphology, chemical structure, and relative molecular weight.

Notably, inter-study discrepancies in secondary-aging performance evolution can be attributed to differences in bitumen source, rejuvenator type and dosage, and aging protocols. For instance, bitumen from different sources exhibits distinct colloidal structures and aging sensitivity, directly affecting degradation patterns. As also described in Section 3.1 and Section 3.2, varying the rejuvenator’s chemical composition leads to different rejuvenation effects and aging resistance on RB. This causes the secondary-aging performance variation in RB in one study to differ from that of RB in others. In particular, aging duration and pressure critically determine long-term aging extent and resistance. Accordingly, RB aging resistance and its influencing factors will be systematically investigated next. Most existing studies focus only on the correlation between asphaltene content and RB performance. The impact of post-rejuvenation colloidal stability on long-term performance remains understudied. Fewer studies focus on micromorphology and LMS before/after secondary aging than on chemical structures. A macro–micro evaluation framework for secondary-aging effects has not yet been developed, thus preventing clear elucidation of RB secondary-aging mechanisms.

### 4.3. Aging Resistance

#### 4.3.1. Aging Resistance of Rejuvenators

Rejuvenator aging resistance has a direct critical impact on secondary-aged RB performance; rejuvenator type affects the aging resistance of RB [178]. In general, better rejuvenator aging resistance correlates with stronger aging resistance of the resulting RB [171,176,179]. To deepen our understanding of this correlation, intrinsic rejuvenator properties and structures are investigated in selected studies to uncover the source of their aging resistance. Effects of inherent properties of different rejuvenators on aging resistance are summarized in Table 6. Notably, neat rejuvenator performance does not reflect RB performance, as rejuvenator chemical properties change during the rejuvenation process [82]. Direct evaluation is typically performed via residual mass retention tests, thermogravimetric analysis, and gas chromatography–mass spectrometry. It is widely recognized that rejuvenators with high molecular weight, good thermal stability, and low volatility generally exhibit better anti-aging performance [76,171,174,197]. Besides direct tests, indirect methods are also used by researchers to assess rejuvenator aging resistance. These methods typically compare the amplitude of performance variation in different rejuvenators during aging. For instance, Xu et al. [201] investigated rejuvenator aging resistance based on rheological and chemical property variation amplitude of two bio-oils and two WEOB under different aging conditions. They found that WEOB aging resistance outperforms that of bio-oils. CI and SI growth amplitude of rejuvenators under different aging conditions is studied by Abdelaziz et al. [115]. Their results confirm that both petroleum-based and bio-based rejuvenators have favorable aging resistance.

#### 4.3.2. Aging Resistance of RB

**Figure 9:**
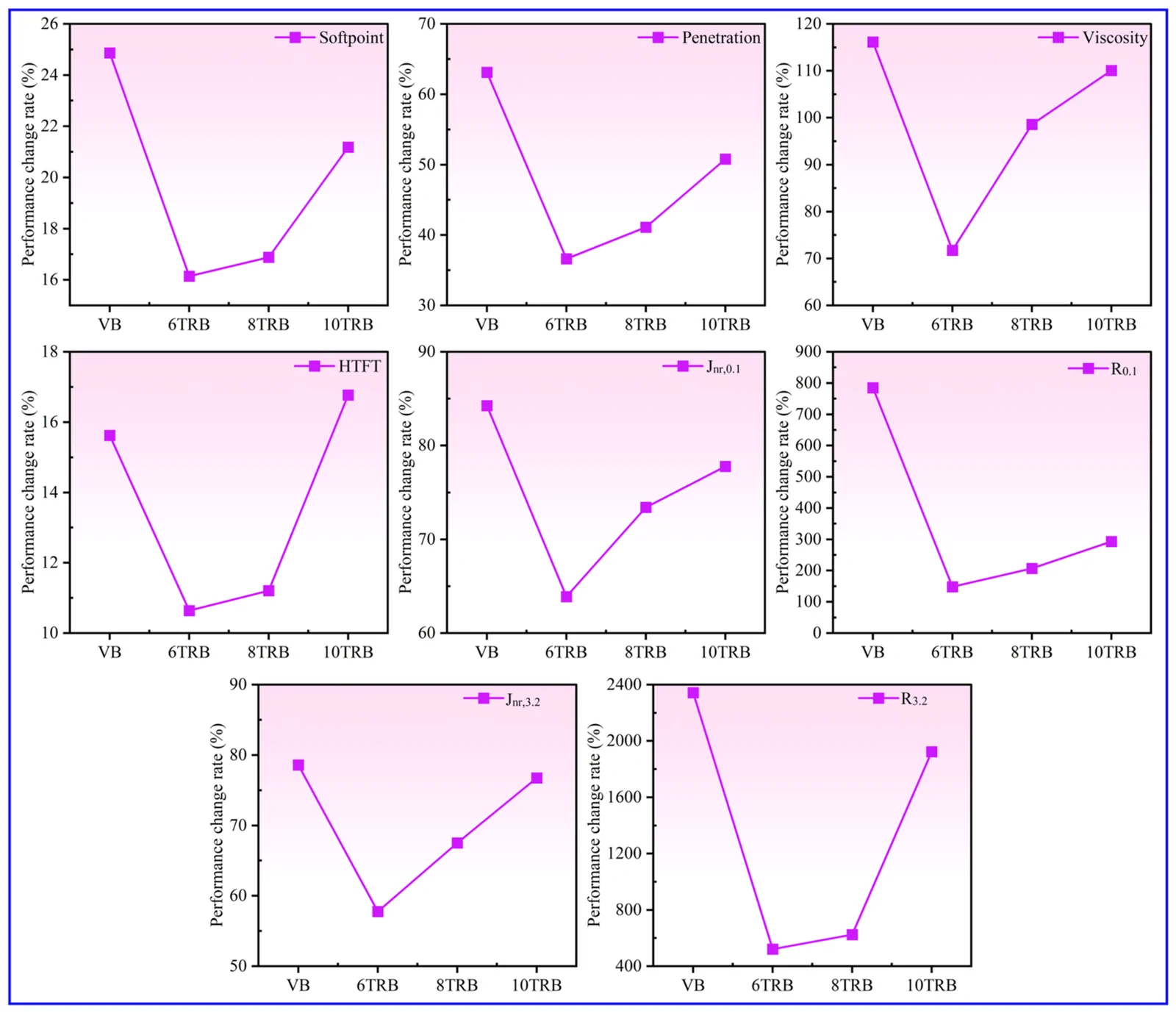
The aging resistance results of RBs (different dosages) and VB; these data were obtained from Reference [13].

To further investigate rejuvenator effects on RB aging resistance, secondary-aging property-variation amplitudes of VB and RB are compared to clarify RB aging resistance across different performance indicators. Figure 9 and Table 7 present and summarize the aging resistance of RB and VB across different performance indicators, respectively. As illustrated, long-term aging resistance of RB prepared with the same rejuvenator and equal dosage varies across properties, with notable discrepancies observed [104,181,191]. Among the data compiled in Figure 9, the property variation amplitudes of softening point and HTFT are the lowest, i.e., approximately 10~20%. This indicates that the aging resistance of RB and VB under these two indicators is superior to that under other indicators. The property variation amplitudes of penetration, viscosity, and *J_nr_* fall within an intermediate range of approximately 60~120%. In contrast, the property variation amplitude of R is far higher than that of the other indicators, exceeding 150% at 0.1 kPa stress and 400% at 3.2 kPa stress. This suggests that the aging resistance of RB and VB under the R indicator is the poorest relative to that under all other evaluated indicators.

The data summarized in Figure 9 and Table 7 also demonstrate that rejuvenator dosage exerts a significant influence on the aging resistance of RB. For instance, studies by Liu et al. [27] and Zhu et al. [13] show that RB aging resistance declines with rising rejuvenator dosage. Other studies show that RB performance recovery after rejuvenation increases secondary-aging sensitivity. Rejuvenator light components in RB are highly affected by aging and more susceptible to oxidation [29,92]. Differentiated impacts of rejuvenator dosage on the aging resistance of RB across different properties are also identified in studies by Yan et al. [99] and Geng et al. [167]. Overall, RB aging resistance declines with increasing rejuvenator dosage, and a balance point exists between rejuvenation efficacy and aging resistance. Notably, studies by Liu et al. [27] and Zhang et al. [200] also find that the negative effect of rejuvenator dosage on RB aging resistance gradually weakens with longer aging time.

Based on the above review, rejuvenator dosage and inherent aging resistance are recognized as critical factors influencing the aging rate of RB, which corroborates conclusions in Section 4.1. This shows that rejuvenator dosage must be properly regulated to balance overall rejuvenation efficacy across all properties and aging resistance of RB. Due to diverse rejuvenator material properties, structures, and complex chemical compositions, in-depth understanding of inherent aging resistance for different rejuvenators has not yet been developed. Meanwhile, when RB and VB aging resistance are compared by relevant properties in existing studies, initial performance consistency of rejuvenated AB is overlooked. In this review, aging resistance comparisons are conducted within each individual study to minimize cross-study variability. Even so, the initial performance levels of VB and RB should ideally be sufficiently comparable to ensure a fairer evaluation, as aging sensitivity may vary nonlinearly across different performance ranges. Moreover, owing to the diversity of bitumen performance indicators, it is practically difficult to achieve full equivalence across all properties, and comparability can therefore only be prioritized based on key properties.

## 5. Scheme Development and Performance Evolution of Bitumen Under Multiple Rejuvenation Cycles

### 5.1. Scheme Development of Bitumen Under Multiple Rejuvenation Cycles

In global asphalt pavement rejuvenation research, primary AB rejuvenation and utilization studies are well established and are widely recognized as a key strategy for environmental protection and resource conservation. Rejuvenated mixtures are currently used mainly for low-grade road surface courses and pothole repair, and frequently serve as base or subbase courses for high-grade pavements [9,206]. As RB secondary-aging research advances, concepts of secondary rejuvenation and further rejuvenation cycles have been gradually proposed in recent years. These concepts are grounded in three main considerations, including engineering applications, theoretical development, and technological progress.

For engineering applications, primary rejuvenation has been widely implemented in road engineering [32]. Nevertheless, rejuvenated pavements inevitably undergo secondary aging during service under combined traffic loading and environmental factors. When these rejuvenated pavements reach the end of their service life and undergo rehabilitation, the secondary RAP (i.e., RAP reclaimed from rejuvenated pavements) will be generated. If these secondary RAP materials are not further recycled, the resource and environmental benefits initially achieved through primary rejuvenation would be partially compromised. As primary-rejuvenation practices continue to expand globally, the cumulative volume of secondary RAP is expected to grow accordingly. This renders multiple rejuvenation cycles an inevitable trend in sustainable asphalt pavement management. Road management authorities have also started prioritizing asphalt pavement recyclable cycle counts [207]. Pasetto et al. [33], Hugener et al. [31], and Porot et al. [208] all agree that RB is currently widely used in road engineering. The continuing expansion of RAP incorporation suggests that multiple bitumen rejuvenations will likely become routine in the future.

For theoretical development, the knowledge framework established for primary rejuvenation cannot be directly extrapolated to multiple rejuvenations. Complex interactions occur between VB, AB, and rejuvenators, and microscopic mechanisms and macro–micro performance-degradation rules of multi-rejuvenated pavements remain unclear [209]. Relevant studies report a cumulative effect of AB and rejuvenators with increasing rejuvenation cycles that significantly affects RB stress relaxation performance [94,210]. Hugener et al. [31] further note that rejuvenators accumulate steadily across multiple bitumen-rejuvenation cycles. Once a critical concentration is reached, they may have uncharacterized effects on pavement performance. These cumulative and interactive effects distinguish secondary and subsequent rejuvenation from primary rejuvenation and render the underlying mechanisms far more complex. Accordingly, RB long-term performance and multiple-rejuvenation efficacy issues must be prioritized for research [33,211]. For technological development, two core challenges are being explored by researchers. The first is how many aging–rejuvenation cycles current rejuvenators can support while continuously restoring key AB properties after each aging cycle [183]. The second is how to resolve compatibility degradation between VB and AB caused by aggregate surface component migration from repeated physicochemical interactions [212,213].

Overall, asphalt pavement multi-rejuvenation technology is more complex than primary-rejuvenation technology. Its complexity stems from both integrated construction processes and multi-dimensional nonlinear material degradation mechanisms. This poses significant challenges to technical applicability evaluation and design standard development for multi-rejuvenated pavements [209]. Driven by worsening service environments and growing traffic volumes on rejuvenated pavements, secondary aging and further aging issues will become increasingly prominent. Therefore, research on bitumen secondary and further rejuvenation is expected to remain a high-priority topic for the foreseeable future.

### 5.2. Evolution of Technical Properties of Bitumen Under Multiple Rejuvenation Cycles

Currently, field-scale engineering practices of multiple rejuvenation are scarcely documented, primarily due to the implementation challenges and prolonged time scales associated with multi-cycle recycling. Accordingly, existing studies on multiple rejuvenation are predominantly conducted under laboratory-simulated conditions. Building on prior studies of primary aging, primary rejuvenation, and secondary bitumen aging, secondary rejuvenation causes predictable changes in conventional technical properties. These include lower softening point and higher penetration and ductility. For rheological properties, G*, G*/sinδ, and G*·sinδ decline, while δ rises. Notably, key performance indicators can be restored to VB baseline levels [94,183,186,207]. As cycle number increases, bitumen’s viscoelastic transition temperature rises monotonically. Low-strain *N_f_* is increasingly dominated by the cumulative superposition of aging and rejuvenation effects [183,187]. Bitumen’s technical and rheological performance after secondary aging or secondary rejuvenation is poorer than that after primary aging, and lower than unaged VB [99,167,179,181,183,187].

**Figure 10:**
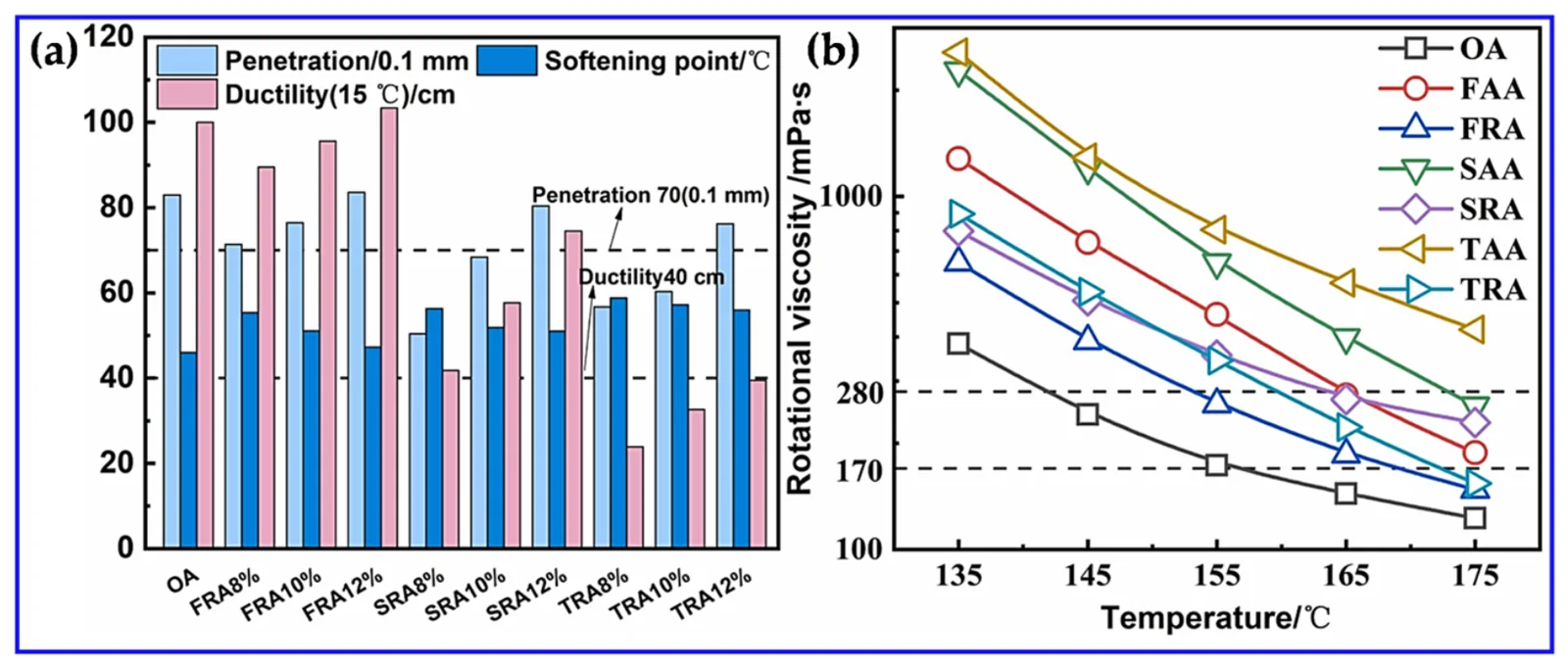
Basic performance results of bitumen subjected to multiple laboratory-simulated rejuvenation cycles ((**a**) three major parameter results and (**b**) viscosity result) [25].

**Figure 11:**
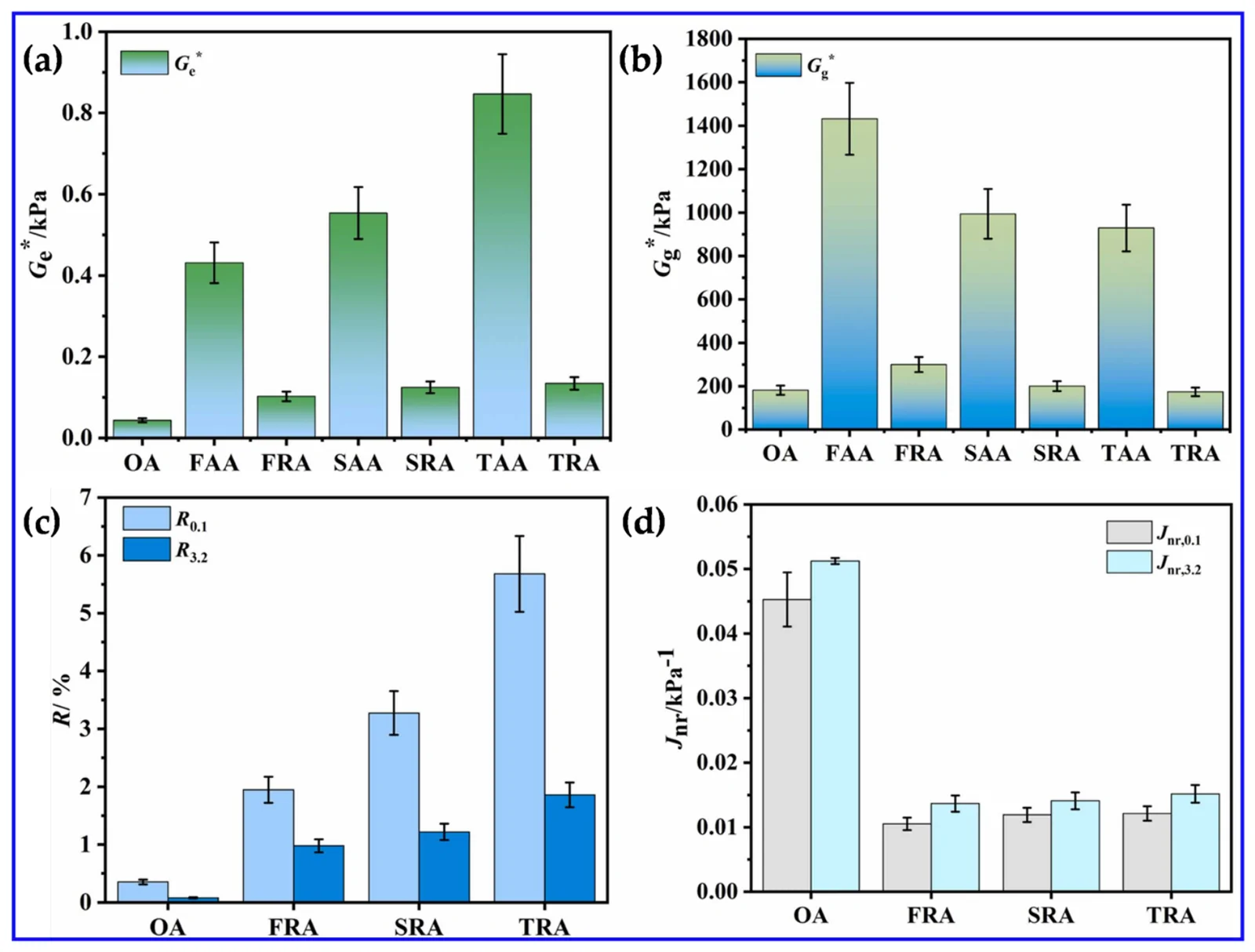
Rheological performance results of bitumen subjected to multiple laboratory-simulated rejuvenation cycles ((**a**,**b**) frequency sweeping result and (**c**,**d**) MSCR result) [25].

Preliminary studies on performance evolution across extended aging–rejuvenation cycles have been published in recent years. Consistent findings show that overall evolution patterns per rejuvenation cycle follow the same trend as primary rejuvenation, as established in Section 4.2. Figure 10 and Figure 11 present the basic and rheological properties of bitumen under laboratory-simulated multiple rejuvenation cycles, respectively. Most properties exhibit progressive deterioration after each aging and rejuvenation cycle. The progressive deterioration trends of softening point, penetration, and viscosity are relatively insignificant, whereas those of ductility and the four rheological indicators are markedly pronounced. Notably, with optimized rejuvenation strategies, selected performance indicators can be restored to VB baseline levels [32,33,77,175]. It has been reported that conventional bitumen technical properties can be restored to pre-aging levels even after five consecutive aging–rejuvenation cycles [214].

Table 8 summarizes existing studies on the technical performance of bitumen under multiple laboratory-simulated rejuvenation cycles. Data in the table show that secondary RB achieves measurable performance recovery. Its recovery magnitude is, however, lower than that of primary rejuvenation, with irreversible property degradation. For extended aging–rejuvenation cycles, existing research confirms that cycle count and rejuvenator dosage are key determinants of final bitumen performance. Technical properties degrade progressively with increasing cycle number. However, cumulative effects of multi-component accumulation and their underlying mechanisms remain unclear. Standardized rejuvenation evaluation indicators tailored for multiple rejuvenations remain limited to date. Therefore, dedicated evaluation metrics for repeated-cycle bitumen rejuvenation are urgently needed. Furthermore, research on multiple aging and rejuvenation remains limited. Most studies cover a maximum of three cycles. Deeper studies are therefore warranted to examine performance evolution at higher cycle counts.

### 5.3. Evolution of Physicochemical Performance of Bitumen Under Multiple Rejuvenation Cycles

**Figure 12:**
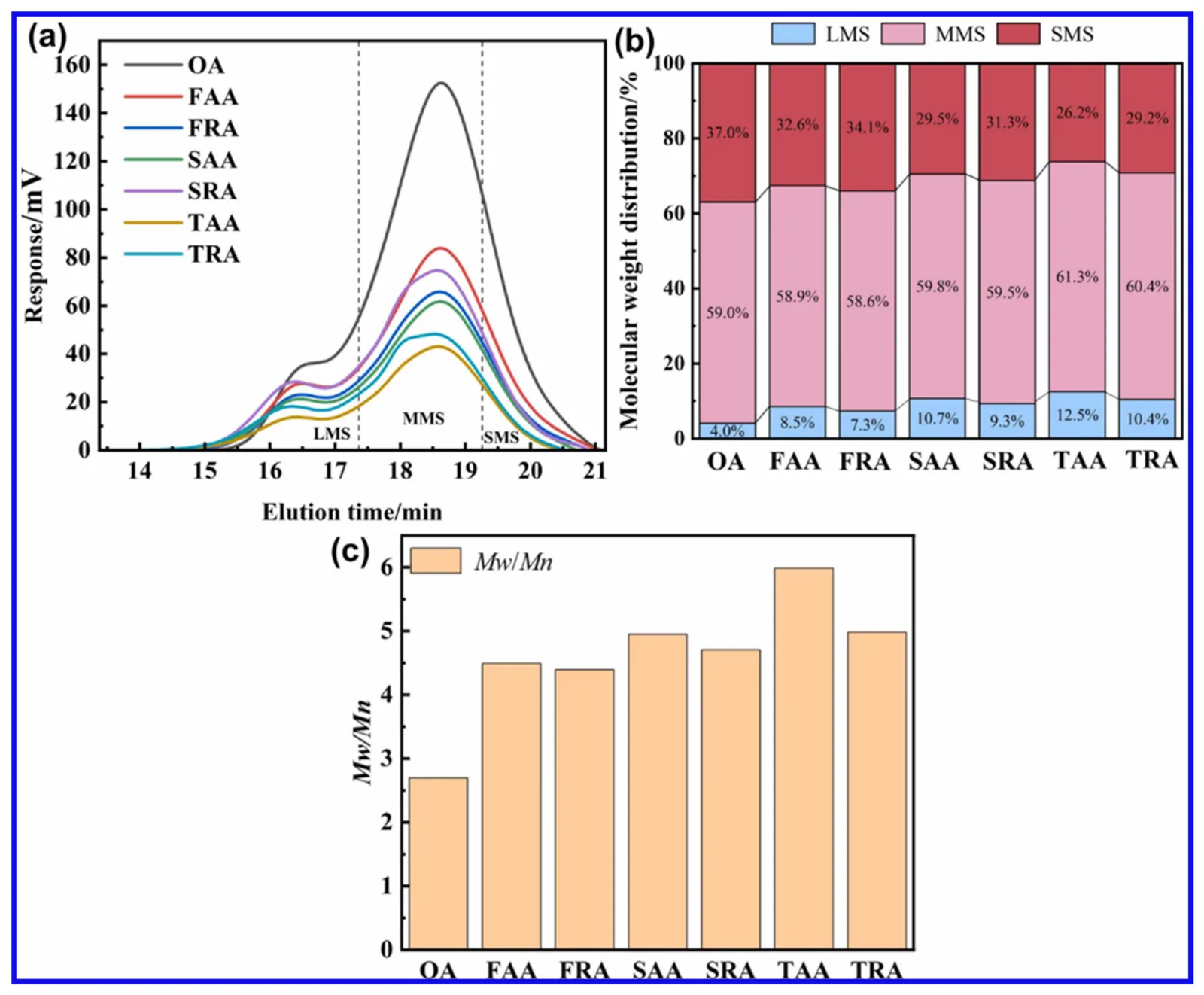
GPC results of bitumen after multiple laboratory-simulated rejuvenation cycles ((**a**) response; (**b**) molecular weight distribution; (**c**) M_w_/M_n_) [25].

**Figure 13:**
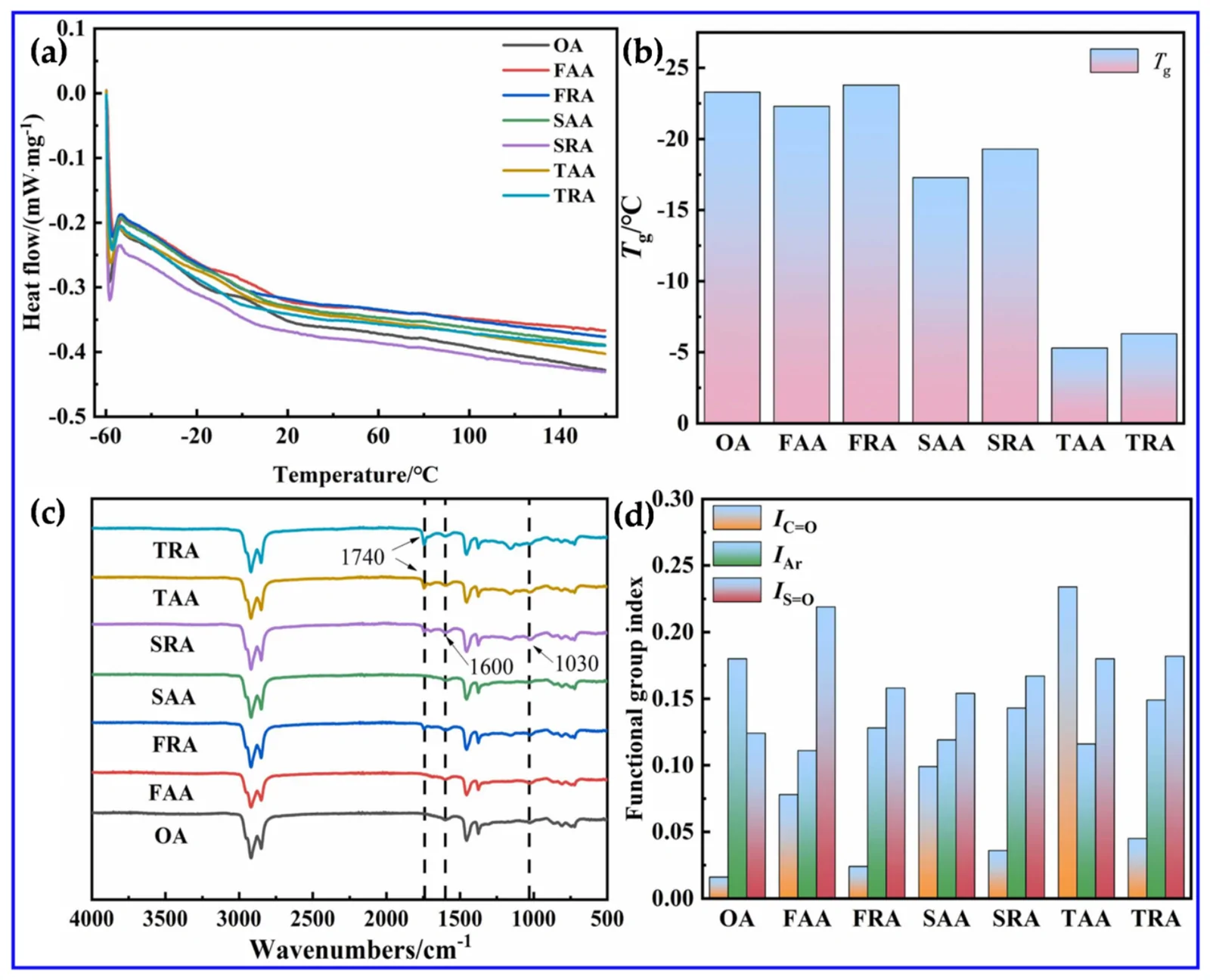
DSC and FTIR results of bitumen after multiple laboratory-simulated rejuvenation cycles ((**a**,**b**) DSC result and (**c**,**d**) FTIR result) [25].

Figure 12, Figure 13, and Table 9 present bitumen physicochemical performance results and property evolution patterns across multiple laboratory-simulated rejuvenation cycles. As indicated by the figures and table, physicochemical indicators of RB, including CI, SI, M_W_, and surface roughness, all increase with cycle number. They exceed values from the prior rejuvenation cycle. This trend aligns with conclusions from multiple bitumen-rejuvenation technical-property studies. The AFM-based conclusion of Chen et al. [32] is further verified by the study of Yu et al. [146]. They report that rejuvenation increases AB molecular mobility and promotes polar component accumulation and nucleation, resulting in larger and taller bee-like structures. This finding further corroborates the earlier conclusion that bitumen’s physical properties degrade progressively with each aging–rejuvenation cycle as total cycle count rises. Similarly, studies on microscopic mechanisms of rejuvenator–bitumen cumulative effects and interactions across repeated cycles remain scarce. Overall, research on multiple bitumen rejuvenations remains limited, with even fewer studies focusing on underlying mechanisms and characterization methods. Diverse research methods need to be developed in future work to establish a systematic evaluation framework for multiple-rejuvenation performance.

## 6. Conclusions

Focusing on the full life cycle of bitumen aging and rejuvenation cycles, this review systematically elucidates primary-aging mechanisms, rejuvenator classification, and associated rejuvenation mechanisms; summarizes secondary-aging performance-attenuation patterns and key aging resistance determinants; and analyzes performance evolution trends across multiple rejuvenation cycles. Based on the evidence compiled from the existing literature review, the following conclusions are derived.

(1) Bitumen thermo-oxidative, ultraviolet, and moisture aging mechanisms differ significantly. Component variations induced by the same aging type can show minor discrepancies, while associated performance changes are largely consistent. Rejuvenator effects on AB are primarily achieved via asphaltene penetration, softening, and dilution. Among petroleum-based rejuvenators, those with an aromatic content above 60% are widely preferred, as the aromatic fractions in such rejuvenators exhibit high dissolving capacity for aged asphaltenes.

(2) Petroleum-based aromatic oils exhibit a density of approximately 1.05 g/cm^3^ and a solubility parameter of 19 J/cm^−3/2^. Both are comparable to those of AB, thereby ensuring favorable compatibility with AB. Triglyceride/fatty acid-based and tall oil-based rejuvenators demonstrate good comprehensive rejuvenation performance. However, existing studies frequently misapply molecular models of FFA to represent triglyceride-based rejuvenators. This may lead to an erroneous understanding of the rejuvenation mechanisms of triglyceride-based bio-oils at the molecular scale.

(3) The variation trends of technical and physicochemical properties of VB and RB before and after aging confirm that RB exhibits poorer sensitivity to secondary aging. The aging resistance of most RB is significantly higher than that of VB. The property variation amplitudes of softening point and HTFT after aging remain within approximately 20%, whereas those of penetration, viscosity, and *J_nr_* are in the range of approximately 60~120%. Macro- and micro-property variation trends of secondary-aged RB are generally consistent with those of bitumen during primary aging.

(4) The aging resistance of RB is both strongly correlated with rejuvenator inherent aging resistance and closely associated with applied rejuvenator dosage. Excessive rejuvenator dosage impairs both RB performance and aging resistance, even if the rejuvenator itself has favorable anti-aging properties. The balance between RB rejuvenation efficacy and aging resistance should be prioritized when AB is rejuvenated via rejuvenator addition.

(5) Bitumen performance variation across multiple rejuvenation cycles is largely consistent with that during primary rejuvenation. The progressive bitumen performance degradation after each cycle is observed with increasing cycle count, which is attributed to raw material cumulative effects. In other words, technical performance is poorer than that of the prior cycle. Bitumen physicochemical indicators after each cycle, including micro-scale surface roughness, CI, and SI, are all higher than those of the preceding cycle.

Overall, this review provides a systematic synthesis of the performance evolution and underlying mechanisms of RB across secondary aging and multiple rejuvenation. It thereby fills a critical gap in the existing literature. These findings offer theoretical support for the rational design and sustainable multiple rejuvenation of RAP resources.

## 7. Future Research Perspectives

Bitumen rejuvenation delivers significant economic and environmental benefits, but multiple challenges remain in the rejuvenation process and long-term service. Key future research directions are outlined as follows:

(1) Current macro and micro testing methods for bitumen aging and rejuvenation cycle studies are inadequate. Diverse macro–micro methodologies should be developed to support multiple aging and rejuvenation research. A robust evaluation system for rejuvenation performance and aging resistance should be established.

(2) Current rejuvenator dosage determination relies solely on bitumen’s physical properties. Future studies can optimize rejuvenator dosage from additional macro–micro performance perspectives to improve the aging resistance of RB. Rejuvenators can be categorized by aging resistance, and new composite anti-aging rejuvenators should be further developed to deliver comprehensive long-term aging resistance across all performance dimensions and ensure good long-term RB service performance.

(3) For developing high-performance bitumen from AB feedstock, rejuvenation strategies focused solely on performance parity with VB should not be overprioritized. Given the diversity of existing bio-oil molecular models, a standardized characterization system for bio-oil fatty acid profiles needs to be established.

(4) The mechanism by which raw material cumulative effects affect bitumen performance per cycle during multi-rejuvenation remains complex and requires further investigation. Meanwhile, bitumen interfacial properties and rejuvenator diffusion behavior per rejuvenation cycle become more complex due to raw material accumulation and multi-component interactions. Relevant studies can be further conducted using molecular dynamics simulations in future work.

## Figures and Tables

**Table 1:** The action mechanisms of various aging types and their effects on bitumen performance.

Aging Type	Action Mechanism	Component Variation				Chemical Structure	Technical Performance	References
		Asphaltenes	Saturates	Aromatics	Resins			
Thermo-oxidative aging (short-term)	Volatilization of light bitumen components occurs at high temperatures, and a small portion of components undergo dehydrogenation to form olefins.	↑	→	↓	↑	Small molecules undergo initial oxidative polymerization into macromolecules, with a small amount of polar functional groups such as carbonyl and sulfoxide groups formed.	Viscosity ↑, penetration ↓, softening point ↑, ductility ↓, rutting resistance ↑, low-temperature cracking resistance ↓	[66,67]
		↑	Slight increase	↓	↓			[35]
Thermo-oxidative aging (long-term)	Continuous oxidation induces further crosslinking of macromolecules, and asphaltenes aggregate to form nano-scale aggregates.	↑	↓	↓	↑	Macromolecular asphaltenes form crosslinked structures via polar functional groups, leading to increased molecular weight and significantly elevated contents of carbonyl and sulfoxide groups.		[13,26,67,68]
		↑	→ or slight increase	↓	↓			[35,66,67]
		↑	Slight decrease	↓	↓			[66,67,69]
Ultraviolet aging	Ultraviolet radiation triggers photo-oxidation reactions, breaks molecular chains, generates free radicals, and accelerates the oxidation and crosslinking of bitumen.	↑	↓	↓	↑	Increased carbonyl and sulfoxide groups, elevated molecular weight, vertical diffusion of macromolecules, and enhanced intermolecular crosslinking.	Rapid surface hardening, brittleness ↑, ductility ↓, fatigue resistance ↓, adhesion ↓	[58,59,70]
		↑	↓	↓	↓			[34]
Moisture aging	Physical diffusion and chemical hydrolysis of water promote coupled thermo-moisture aging and coupled ultraviolet–moisture aging.	↑	↓	↓	↑	Increased carbonyl and sulfoxide groups, aggregation of polar molecules, and destruction of colloidal structure.	Moisture stability ↓, rheological properties ↓, adhesion ↓, fatigue life ↓	[60,62]
		↑	Maltenes ↓					[60,71]

Note: ↑ indicates increase, ↓ indicates decrease, and → indicates stable.

**Table 2:** Proportions of four components in the rejuvenator.

Rejuvenators	Proportion of Components (%)				References
	Saturates	Aromatic	Asphaltene	Resin	
Aromatic oil	-	90.79	-	-	[72]
	-	78	-	-	[95]
	-	>80	-	-	[88]
Naphthenic oil	85.53~91.76	6.02~11.18	0~0.45	-	[96]
WEO	24.8	61.7	-	-	[27]
Plant extract	0.44	72.49	0.71	26.36	[69]
	1.45	89.16	0.82	8.57	
Vegetable oil	2.74	92.86	0.33	4.11	
Aromatic extract	20.56	72.82	0.25	6.37	
RA100	22.5	65.8	-	-	[97]
RA102	19.2	71.0	-	-	
WCO	31.6	63.6	-	4.8	[98]
WBO	22.4	54.2	0.9	22.5	
WEO *	4.9	63.2	3.7	24.3	
WCO	0	70.5	0	29.5	[99]
Tall-oil fatty acids	0	83.02	12.58	4.40	[13]
Commercial rejuvenator	24	30.25	5.1	40.65	[100]
Bio-derived rejuvenator	19.5	40.25	4.32	35.95	
Bio-rejuvenator	5	3	39	53	[101]
Bio-oil	10.12	68.25	10.17	11.46	[8]
Swine-based rejuvenator	1.93	3.06	23.76	71.24	[102]
Algae-based rejuvenator	0	15.37	1.13	83.48	
WCO	30.52	38.66	3.46	27.36	[103]
WEO	27.5	4.68	11.50	56.32	
Agriculture-based rejuvenator	-	-	-	100	[104]
Petroleum-based rejuvenator	9.5	84.0	-	6.5	

Note: * indicates a 3.9% ash content.

**Table 3:** Molecular models adopted for bio-oils.

Rejuvenator	Molecular Name or Molecular Formula	Form	References
Epoxidized soybean oil, WCO	C_18_H_32_O_2_	Free	Long et al. [14]
WCO	Palmitoleic acid, oleic acid, stearic acid		Aljarmouzi et al. [156]
	Hexadecanoic acid, linolenic acid, stearic acid, oleic acid		Ji et al. [157]
	Linoleic acid, palmitic acid, oleic acid		Yu et al. [158]
	Linoleic acid, palmitic acid, oleic acid		Wang et al. [111]
	C_21_H_38_O_4_, C_18_H_34_O_2_, C_16_H_32_O_2_, C_14_H_28_O_2_		Shi et al. [110]
	Linolenic acid, palmitic acid, oleic acid		Xu et al. [159]
Waste vegetable oil (low acid value)	Linoleic acid, oleic acid		Ruan et al. [155]
Soybean oil	C_18_H_34_O_2_, C_18_H_32_O_2_		Zhang et al. [160]
Tall oil	Abietic acid, linoelaidic acid, oleic acid		Ye et al. [107]
	Palmitic acid, oleic acid		Zhu et al. [15,28]
	Abietic acid, linoleic acid, oleic acid		He et al. [53,108,161]
Soybean oil, rapeseed oil	Tripalmitin, triolein	Triglyceride	Zhu et al. [15,28]
Soybean oil	C_57_H_100_O_6_, C_57_H_98_O_6_, C_55_H_98_O_6_		Ye et al. [107]
Vegetable oils	Trilinolenin, tripalmitin, triolein, trilinolein		Xiong et al. [162]
WCO	Tripalmitin, triolein, trilinolein		Yan et al. [152,153,154]
Treated WCO	Methyl linoleate, methyl stearate, methyl palmitate, methyl oleate	Methyl ester	Zheng et al. [16,17]
Bio-oil	Methyl palmitate, methyl oleate, methyl stearate, methyl eicosenoate, methyl linoleate, methyl elaidate		Ren et al. [109]

**Table 4:** Rejuvenation mechanisms induced by different rejuvenators and their impacts on the macroscopic and microscopic properties of bitumen.

Rejuvenation Mechanism	Mode of Action	Rejuvenators	Molecular Structure Changes	Performance Impact	References
Component supplement	Supplement light components (especially aromatics), restore colloidal structure.	Aromatic oil, aromatic blends, bitumen, bio-oil, WEO	Colloidal structure restored via component supplementation and dilution.	Decreased viscosity and high-temp. performance; improved ductility and rheology, improved construction workability	[47,78,163,164]
Physical dissolution	Rejuvenator penetrates, softens and dilutes asphaltenes.	WCO, bitumen, light oil components, aromatic derivatives	Macromolecules dissolved and dispersed; asphaltene aggregation relatively reduced.		[94,99,163,165,166,167,168,169]
Chemical depolymerization	Integrates asphaltenes; occupies aggregation voids to prevent self-association; promotes depolymerization and peptization.	WCO, bio-oil (better with phenols), tall oil	Macromolecular depolymerization; reduced molecular size distribution and molecular weight.		[53,101,108,170,171]
Molecular lubrication	Small rejuvenator molecules intercalate between bitumen molecular chains, lubricating macromolecules.	Cashew nut-shell oil, corn oil, bio-oil	Increased intermolecular-chain spacing; decreased activation energy.		[165,169]

**Table 5:** The preparation methods of AB and RB adopted in multiple cycles by researchers.

Reference	Rejuvenator	Rejuvenator Dosage	VB	Primary-Aging Process	Primary-Regeneration Process				Secondary-Aging Process	Multiple-Rejuvenation Process	Multiple Aging Process
					Mixed Bitumen Material	Shear Rate (rpm)	Shear Temperature (°C)	Shear Time (min)			
Xu et al. [78]	SMB	100%	-	-	SMB + RAP binder	-	165	5	RTFOT 0, 40, 85, 180, 270 min	-	-
Geng et al. [167]	Composed of light oil, styrene–butadiene rubber (SBR), and liquid rubber	4%, 6%, 8%	Crumb rubber modified bitumen	RTFOT + PAV 20 h	Lab-aged Crumb rubber modified bitumen	5000	180	10	135 °C, Oven 4, 12 h	-	-
Zhao et al. [171]	WCO	3.3%, 3.6%, 5.2%	Pen 60-80 bitumen	TFOT 5 h + PAV 20 h	Lab-AB	2000	165	25	TFOT 5 h + PAV 20 h	-	-
Li et al. [177]	A liquid rich in aromatics	4%	SMB	RTFOT 85 min + PAV 74 h	Lab-aged SMB + VB + SBR	500~1000	170	35	RTFOT 85 min + PAV 0, 6, 16, 28, 42 h	-	-
Cavalli et al. [178]	Bio-based	5%	-	-	RAP binder	3500	-	1	RTFOT + PAV	-	-
Luo et al. [98]	WCO, waste bio-oil, WEO	8% WCO, 10% waste bio-oil, 12% WEO	-	-	RAP binder	4000	-	15	RTFOT + PAV 20, 40 h	-	-
Li et al. [174]	Recycled engine oil bottom	7%	PG 70-28 bitumen	TFOT + PAV	Lab-AB	4000	150	10	TFOT 5, 10, 15, 20, 25 h	-	-
Cao et al. [179]	Corn oil, WEO, vacuum stream oil, cashew shell oil	5%	SMB	RTFOT + PAV	Lab-aged SMB	-	150	20	RTFOT + PAV	-	-
Abdelaziz et al. [115]	Bio-oils, vegetable oils, tall oils, aromatic extracts, paraffin oils	-	PG 64-22 bitumen	-	RAP binder + VB	Hand	160	0.5	RTFOT + PAV	-	-
Masad et al. [180]	REC	1.1%, 1.8%	Pen 60-70	-	RAP binder + VB	150	-	7	RTFOT + PAV 20 h	-	-
Chen et al. [181]	VB	233.33%	-	-	Lab-AB	500 ± 50	140 ± 5	5	RTFOT + PAV	-	-
Liu et al. [27]	WEO	3%, 6%, 9%	70 grade bitumen	RTFOT + PAV	Lab-AB	10,000	-	40	85 °C, Oven 0, 2, 5, 10, 20 d	-	-
Ma et al. [182]	Soybean oil residue, castor oil residue, aromatic oil residue	3%	PG 58-28 bitumen	RTFOT + PAV	Lab-AB	-	140	30	PAV 20, 80 h	-	-
Wang et al. [183]	Bio-oil, re-refined engine oil bottom	5% Bio-oil, 10% re-refined engine oil bottom	PG 58–28 bitumen	PAV 40 h	Lab-AB	800	160	60	PAV 40, 80 h	√	-
Lu et al. [94]	HRA-1	12%	70 grade petroleum bitumen	PAV 20 h	Lab-AB + VB	-	155	15	PAV 20 h	√	-
Koudelka et al. [184]	11 rejuvenators	-	50/70 bitumen	RTFOT + PAV 20, 40 h	Lab-AB	-	-	-	RTFOT + PAV 20, 40 h	√	RTFOT + PAV 20 h
Chen et al. [32]	Petroleum-based oil	1st 6%, 2nd 10%, 3rd 14%	SMB	RTFOT + PAV 20 h	Lab-AB	-	140~160	30	PAV 20 h	√	√
Blomberg et al. [175]	650/900 bitumen	-	Pen 70/100 bitumen	RTFOT + PAV 20 h	Lab-AB	-	145	30	RTFOT + PAV 20 h	√	√
Rodrigues et al. [77]	Two bio-oils and a soft binder	-	3045 Pen grade bitumen	RTFOT + PAV60 h	Lab-AB	150	150	15	RTFOT + PAV 60 h	√	√
Nie et al. [185]	Composed of epoxy resin, vegetable oil, 90 grade bitumen	-	AH-70 bitumen	RTFOT 40, 85, 180, 240, 300 min	Lab-AB	10,000	120	30	RTFOT 40, 85, 180, 240, 300 min	√	√
Chen et al. [186]	Grade 60/80 bitumen	233.33%	Grade 60/80 bitumen	TFOT 5 h + PAV 20 h	Lab-AB	-	-	-	TFOT 5 h + PAV 20 h	√	√
Abe et al. [187]	R1, R2	6%	Grade 70/100 and grade 50/70 bitumen	RTFOT + PAV	Lab-AB	500~700	150	20	PAV	√	√

Note: √ indicates consistent with the primary-regeneration process or consistent with the secondary-aging method.

**Table 6:** Effects of inherent properties of different rejuvenators on their aging resistance.

Rejuvenator	Characteristics	Anti-Aging Mechanism	Effect	References
Paraffinic oils	No polar compounds contained.	Strong oxidation resistance, no easily oxidizable sites in molecules, high chemical stability, positive effect on the oxygen balance of AB, and inhibition of continuous aging.	Positive	[82]
Oxidized products of waste-oil residue	Specific oxidation products such as ketones, acid anhydrides, and monosubstituted benzenes.	Exert positive effects on the oxidation balance of AB and inhibit continuous aging.		[47]
Nut-shell oil	Benzene derivatives with p-π conjugated structures.	Capture and stabilize free radicals, presenting favorable anti-aging properties.		[179,202]
Aromatic oils	No easily oxidizable sites in molecules.	High chemical stability.		[82]
Phenolic substances	Contain hydroxyl groups (-OH) and methoxy groups (O-CH_3_).	Provide active sites and improve the anti-aging performance of RB as sacrificial agents.		[102,203]
Crumb rubber/polyethylene composites	Dispersed polyethylene particles interact with rubber to form a network structure.	The network structure enhances the anti-aging performance of bitumen.		[204]
Vacuum residue	Non-paraffinic alkane compounds.	Low initial decomposition temperature and poor aging resistance.	Negative	[179]
Agricultural-based rejuvenators	Contain dialkyl/aryl sulfones and ester groups.	Dialkyl/aryl sulfones and ester groups exert adverse effects on long-term aging performance.		[104]

**Table 7:** The comparison of long-term aging resistance of RB and VB across different properties.

Reference	VB	Mixed Bitumen Material	Rejuvenator	Aging Process	Rejuvenator Dosage	Evaluation Index	Conclusion
Zargar et al. [205]	80/100 grade bitumen	Aged 80/100 grade bitumen	WCO	RTFOT 85 min	1%, 2%, 3%, 4%, 5%	Penetration	R > V
						Viscosity	
						G*	
						Asphaltene content	
Chen et al. [181]	70# matrix bitumen	RAP binder	70# matrix bitumen	RTFOT 85 min + PAV 20 h	233.33%	SI	R > V
						CI	
						T_g_	R < V
						S	
						m-value	
						Viscosity	
						Softening point	
						G*	
Cavalli et al. [178]	50/70 bitumen	RAP binder	Natural seed oil, cashew nut-shell oil, tall oil	RTFOT 85 min + PAV 20 h	5%	G*	R (natural seed oil) > V, R (cashew nut-shell oil, Tall oil) < V
						Crossover temperatures	
						CI	R > V
						Asphaltene content	
Haghshenas et al. [104]	PG 64-34 bitumen	RAP binder + PG 64-34	R1, R2	RTFOT + PAV	23.1%	CI	R > V
						Asphaltene content	R < V
						SI	
Borghi et al. [191]	50/70 bitumen	RAP binder	Pine bio-rejuvenator	RTFOT 85 min + PAV 20 h	12.4%	Penetration	R < V
						Softening point	
						Viscosity	R > V
						Rheological index	
						Crossover frequency	
						Percent recovery (R)	
						*J_nr_*	
						*D_f_*	
Liu et al. [92]	PG 64-22 bitumen	Aged PG 64-22 bitumen	WEO	85 °C Oven 20 d	9%	G*	R < V
						δ	
						G-R	
						CI	
Liu et al. [27]	A 70# bitumen	Aged A 70# bitumen	WEO	85 °C Oven 5 d	3%, 6%, 9%	CI	R (3%, 6%) > V, R (9%) < V
						G*/sinδ	
						Crossover frequency	R (3%) > V, R (6%, 9%) < V
						G-R	
						Crossover modulus	R > V
						Rheological index	
Zhang et al. [200]	A 70# bitumen	Aged A 70# bitumen	WEO	85 °C oven, 5 d	3%, 6%, 9%	CI	R < V
Wang et al. [197]	A 70# bitumen	Aged A 70# bitumen	WCO	RTFOT 85 min + PAV 20 h	6%	LMS	R > V
						M_W_	
Ye et al. [107]	PG 64-22 bitumen	Aged PG 64-22 bitumen	Soybean oil, Aromatic oil, distilled tall oil:cardanol = 7:3	RTFOT 85 min + PAV 20 h	7%	G*	R (Aromatic oil) > V
						δ	R > V
Geng et al. [167]	CRMA	Aged CRMA	Composed of light oil, SBR and liquid rubber	TFOT 5 h + PAV 20 h	4%, 6%, 8%	Softening point	R (4%, 6%) > V, R (8%) < V
						Penetration	R < V
						Viscosity	R (4%) > V, R (6%, 8%) < V
						Ductility	R > V
						G*/sinδ	
Zhao et al. [171]	Penetration 60–80 bitumen	Aged penetration 60–80 bitumen	WCO	TFOT 5 h + PAV 20 h	3.3%, 3.6%, 5.2%	Viscosity	R > V
						G*/sinδ	
						CI	
Yan et al. [99]	90A bitumen	Aged 90A bitumen	WCO	RTFOT 85 min	4%, 8%	G*/sinδ	R > V
						δ	
						CI	
						T_g_	
						LMS	
						*N_f_*	R (4%) < V, R (8%) > V
Mohammadafzali et al. [173]	PG 64-22 bitumen	Aged PG 64-22 bitumen	HPE, CWE	RTFOT + PAV 20 h	Equivalent PG method	S	R < V
						m-value	
Chen et al. [186]	60/80 grade bitumen	Aged 60/80 grade bitumen	60/80 grade bitumen	TFOT 5 h + PAV 20 h	233.33%	Viscosity	R < V
						G*sinδ, G*/sinδ	
						S	
						m-value	
						SI	
						CI	R > V

Note: Rejuvenator dosage is defined as the mass ratio of rejuvenator to AB. The condition where the aging resistance of RB is superior to that of VB is denoted as R > V, while the opposite condition is denoted as R < V.

**Table 8:** Summary of technical performance evolution of bitumen under multiple laboratory-simulated rejuvenation cycles.

Reference	Cycles (Times)	Findings
Hugener et al. [31]	3	The moisture-damage resistance and stiffness properties of bitumen deteriorate progressively after each aging or rejuvenation cycle.
Chen et al. [32]	3	The fatigue life of SMB increases progressively under low stress levels and decreases progressively under high strain levels after each aging or rejuvenation cycle.
Rodrigues et al. [77]	3	A cumulative effect of rejuvenators occurs during multiple rejuvenation cycles that induces excessive softening of RB. The rutting sensitivity of bitumen increases progressively after each rejuvenation cycle.
Xu et al. [215]	3	The recovery efficiency of rheological properties, technical performance, and toughness of SMB decreases progressively, while the viscosity increases progressively after each aging or rejuvenation cycle.
Du et al. [216]	3	The high-temperature performance and viscosity of SMB increase progressively, while the low-temperature cracking resistance, ductility, and elastic recovery rate decrease progressively after each aging or rejuvenation cycle.
Wang et al. [209]	3	During the first two cycles, the degradation of SBS exerts a greater influence on bitumen properties than aging. The *J_nr_* of SMB increases after each aging or rejuvenation cycle.
Cui et al. [25]	3	The high-temperature performance of bitumen increases gradually after each aging or rejuvenation cycle, and the growth amplitude of high-temperature performance becomes more significant with each cycle. The variation amplitude of viscosity exhibits the most prominent progressive increase.
Pasetto et al. [33]	3	The number of rejuvenation cycles or rejuvenator dosage exerts a significant influence on the final properties of bitumen after each rejuvenation cycle.
Gong et al. [211]	4	The relaxation time and relaxation capacity of bitumen decrease progressively after each aging or rejuvenation cycle, and the rejuvenation efficiency of rejuvenators declines accordingly.
Hu et al. [26]	4	The rheological index and low-temperature performance of bitumen decrease progressively, while the fatigue performance increases progressively after each aging or rejuvenation cycle.
Blomberg et al. [175]	4	The content of aromatics and resins in bitumen decreases and increases, respectively, after each aging or rejuvenation cycle, and the rheological properties weaken progressively.
Nie et al. [185]	5	The softening point of bitumen increases progressively after each aging or rejuvenation cycle. The ductility begins to decrease progressively from the third cycle but still meets the specification requirements.

**Table 9:** Summary of physicochemical property evolution of bitumen under multiple laboratory-simulated rejuvenation cycles.

Reference	Cycles (Times)	Findings
Yan et al. [99]	2	Progressive increases in M_W_, CI, and SI of bitumen are observed after each aging or rejuvenation cycle.
Abe et al. [187]	2	The micro-scale surface roughness of bitumen can be restored after each rejuvenation, but the surface roughness of bitumen increases progressively with each aging or rejuvenation cycle as the total number of cycles rises.
Hugener et al. [31]	3	Significant decreases in SI and CI are observed during secondary rejuvenation along with comparable sulfoxide content, indicating favorable overall rejuvenation effects. Complete recovery of SI and CI cannot be achieved via rejuvenation, which leads to a more pronounced secondary-aging effect.
Xu et al. [215]	3	Progressive increases in CI and SI of SMB are detected after each aging or rejuvenation cycle, accompanied by gradually weakening rejuvenator efficacy.
Wang et al. [209]	3	Decreases in the maximum length and roughness of bee-like structures in SMB are observed after each aging or rejuvenation cycle, along with an increase in the number of such structures.
Chen et al. [32]	3	Progressive increases in CI and SI of SMB are noted after each aging or rejuvenation cycle, and butadiene segment content decreases due to oxidative degradation of SBS. Microstructural damage can be restored by rejuvenators. The size of bee-like structures is significantly reduced after the second rejuvenation and significantly increased after the third rejuvenation, respectively.
Du et al. [216]	3	Progressive increases in CI, SI, M_W_, and LMS of SMB are observed after each aging or rejuvenation cycle.
Cui et al. [25]	3	Progressive elevations in T_g_, CI, DMT modulus, and LMS of bitumen are observed after each aging or rejuvenation cycle. The recovery effect of T_g_ of bitumen after the third rejuvenation is inferior to that after the first two rejuvenation cycles. A significant increase in SI is only detected after the first aging cycle, and no obvious change is observed in the subsequent two aging cycles.
Chen et al. [186]	3 agings + 2 rejuvenations	Progressive increases in micro-scale surface roughness, SI, and CI of bitumen are observed after each aging or rejuvenation cycle, accompanied by a progressive decline in the aging resistance of bitumen.
Gong et al. [211]	4 agings + 3 rejuvenations	Progressive increases in the size of bee-like structures and surface roughness of bitumen are detected after each aging or rejuvenation cycle.
Blomberg et al. [175]	4	Progressive increases in resin content and M_W_ of bitumen are observed after each aging or rejuvenation cycle, along with a decrease in aromatic content.
Hu et al. [26]	4	A progressive decrease in aromatic content of bitumen is observed after each aging or rejuvenation cycle, accompanied by progressive increases in asphaltene content and colloidal index.
Schwettmann-Lui et al. [217]	4	Progressive increases in M_W_ and asphaltene content of bitumen are observed after each aging or rejuvenation cycle, along with a consistent shift of the viscoelastic deformation component toward a higher elastic component.
Nie et al. [185]	5	The influence of aging time on performance deterioration is more significant than that of cycle number. Progressive increases in CI and SI of bitumen are observed after each aging or rejuvenation cycle, and the rising amplitude of CI is more pronounced than that of SI.

## Data Availability

No new data were created or analyzed in this study. Data sharing is not applicable to this article.
